# Supplementary material for: Small-RNA-Mediated Genome-wide trans-Recognition Network in Tetrahymena DNA Elimination
Source: Mol Cell. 2015 Jul 16;59(2):229–42. doi: 10.1016/j.molcel.2015.05.024 (PMC4518040; doi:10.1016/j.molcel.2015.05.024)
Supplement: Document S1. Supplemental Experimental Procedures and Figures S1–S3 [file mmc1.pdf]

Molecular Cell

Supplemental Information

Small-RNA-Mediated Genome-wide *trans*-Recognition Network in *Tetrahymena* DNA Elimination

Tomoko Noto, Kensuke Kataoka, Jan H. Suhren, Azusa Hayashi, Katrina J. Woolcock, Martin A. Gorovsky, and Kazufumi Mochizuki

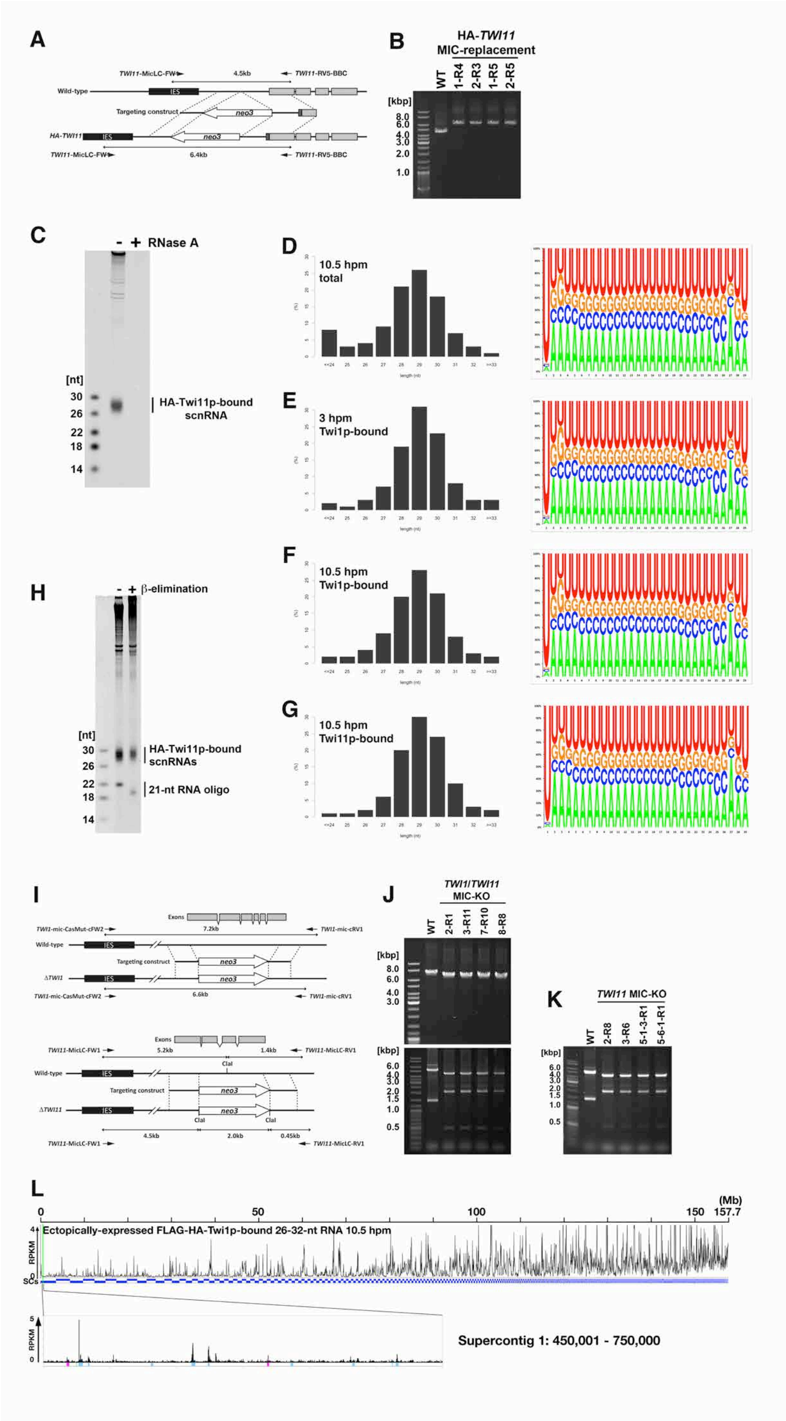

Supplementary Figure S1 (related to Figure 2). Analyses of Twi1p and Twi11p-bound small RNAs.

(A) Diagrams of the wild-type TWI11 and HA-TWI11 MIC loci.
(B) Genomic PCR demonstrating the replacement of endogenous MIC TWI11 genes with the HA-TWI11 construct. Total DNA isolated from the indicated strains was used for PCR with the primers shown in (A). One of the primers was complementary to IESs, and thus, PCR amplified only the loci in the MIC.
(C) HA-Twi11p-containing complexes were immunopurified with anti-HA antibody from the strains in which the endogenous TWI11 loci in the MIC were replaced with HA-TWI11 gene (HA-TWI11) at 10.5 hpm. Co-purified RNAs were incubated with (+) or without (-) RNase A, separated on a denaturing gel and stained with a nucleic-acid specific dye. Positions of single-stranded RNA markers are indicated on the left.
(D-G) Size distributions of total small RNAs (left) and base composition of 29-nt scnRNAs (right) from wild-type cells at 10.5 hpm (D), Twi1p-bound RNAs from wild-type cells at 3 hpm (Early-scnRNAs, E), Twi1p-bound RNAs from wild-type cells at 10.5 hpm (F), and Twi11p-bound RNAs from wild-type cells at 10.5 hpm (Late-scnRNAs, G).
(H) HA-Twi11p-bound RNAs were prepared as in (C), mixed with a synthetic, 21-nt RNA and subjected to periodate oxidation/β-elimination (+). The reactions were separated in a denaturing gel and visualized using a nucleic acid-specific dye. Untreated RNAs (-) were analyzed in the same gel. The positions of the RNA markers are shown on the left. The mobility of the HA-Twi11p-bound small RNAs did not increase after the reaction, whereas the mobility of the unmodified synthetic 21-nt RNA did, indicating that Twi11p-bound Late-scnRNAs were modified at their 3' terminal nucleotides.
(I) Diagrams of the wild-type TWI1 and TWI1 KO (ΔTWI1) loci (top) and the wild-type TWI11 and TWI11 KO (ΔTWI11) MIC loci (bottom).
(J, K) Genomic PCR demonstrating the replacement of endogenous TWI1 and TWI11 genes by the KO constructs in TWI1/TWI11 double MIC-KO (J) and TWI11 (single) MIC-KO cells (K). Total DNA isolated from the indicated strains was used for PCR with the primers shown in (I). For both loci, one of the primers was complementary to IESs, and thus, PCR amplified only the loci in the MIC. PCR products from the TWI11 locus were digested with ClaI.
(L) Normalized numbers (RPKM) of uniquely mapped 26-32-nt RNAs immunopurified with FLAG-HA-Twi1p, which was ectopically expressed from 6 hpm and immunoprecipitated at 10.5 hpm, are shown as histograms with 50-kb (left) or 100-b (right) bins. The 300-kb window on the right is marked with a green line. Colored boxes below the right histograms indicate IES positions (magenta: Type-A, sky-blue: Type-B; see Figure 3 for the IES classification).

Noto et al. Supplementary Figure S1

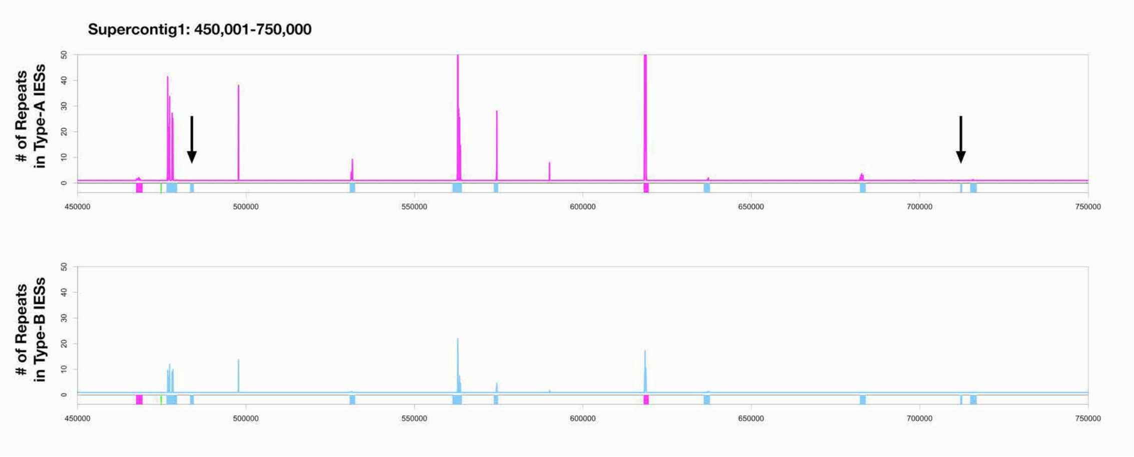

Supplementary Figure S2 (related to Figure 4). Distributions of sequences complementary to IESs.

Distributions of sequences complementary to IESs in a B-region of the MIC genome (300 kb). All possible 25-nt sequences were extracted from all Type-A IESs (magenta, top) and Type-B IESs (sky-blue, bottom). Their occurrence in given genomic locations was calculated and shown as histograms with 100-nt bins. Positions of Type-A and Type-B IESs are marked with magenta and sky-blue boxes, respectively. Type-B IESs possessing no repeat sequences are marked with arrows.

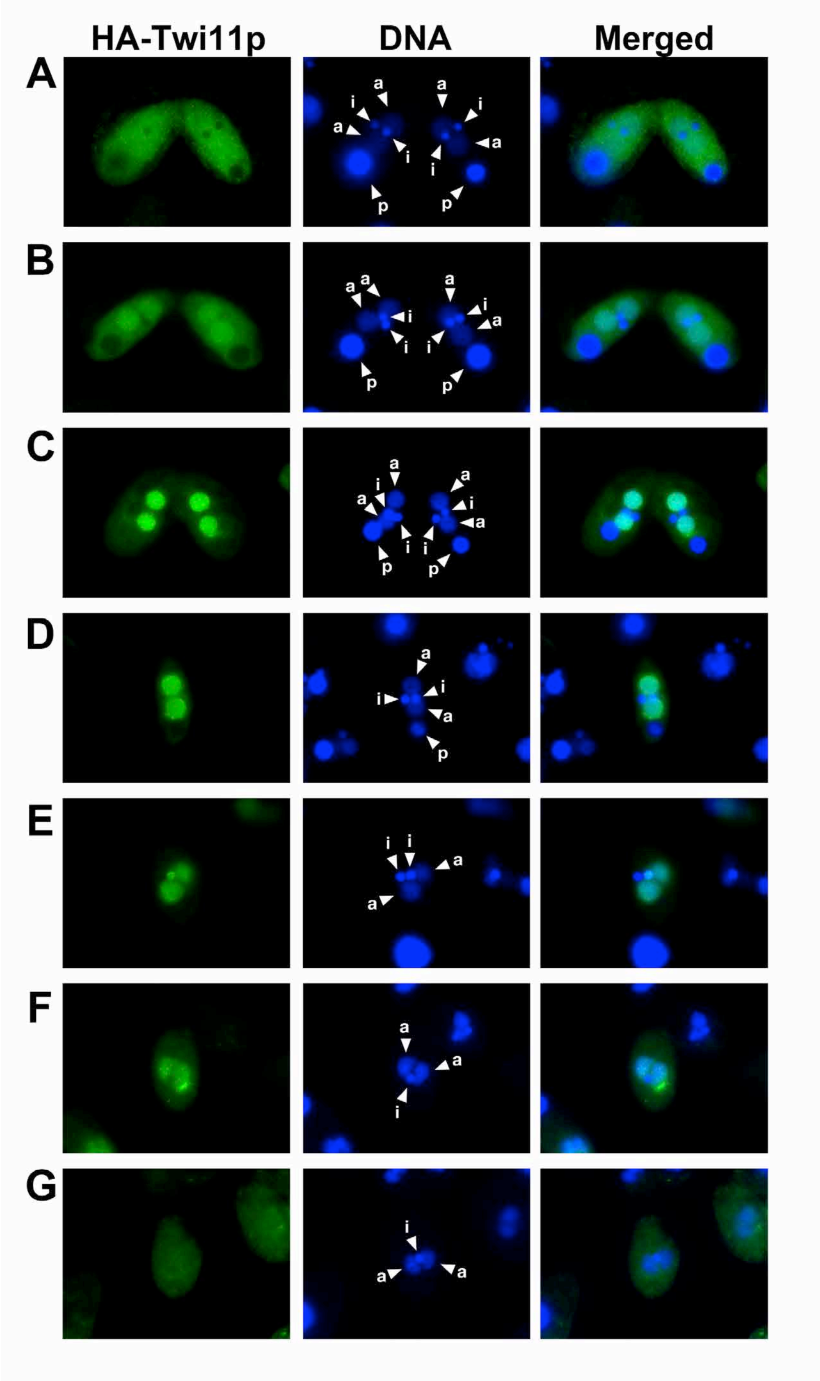

Supplementary Figure S3 (related to Figure 6). Localization of HA-Twi11p.

The localization of HA-Twi11p during late conjugation and exconjugation stages in strains in which the endogenous TWI11 loci in the MIC were replaced with the HA-TWI11 gene (HA-TWI11) was analyzed by indirect immunofluorescent staining using an anti-HA antibody (green). DNA was counterstained with DAPI (blue). Cells were in the Nuclear Alignment stage (A-C) or Exconjugation stage (D-G). HA-Twi11p first appears in the cytoplasm at the Nuclear Alignment stage and then moves into the new MAC, where it remains until the Exconjugation stages. HA-Twi11p also localizes to the MIC during the Exconjugation stage (E), although the significance of this MIC localization is not clear. The parental (old) MACs, the new MACs, and the new MICs are marked by arrowheads labeled “p,” “a” and “i,” respectively.

Supplemental Experimental Procedures

Tetrahymena Strains

Wild-type B2086 and CU428 strains of *Tetrahymena thermophila* were obtained from the *Tetrahymena* Stock Center at Cornell University. The *TWI1* MAC-knockout (KO), *FLAG-HA-TWI1, DCL1* MIC KO, and *EZL1* MAC&MIC KO strains were previously described (Aronica et al., 2008; Mochizuki et al., 2002; Mochizuki and Gorovsky, 2005; Noto et al., 2010). The construction of the *HA-TWI11*, *TWI11* MIC-KO, *TWI1/TWI11* double MIC-KO, *MTT1*::FLAG-HA-*TWI1,* and *RDR1* MIC KO strains is described below. Cells were grown in SPP medium (Gorovsky et al., 1975) containing 2% proteose peptone at 30 °C. For conjugation, growing cells (~5-7 x 10^5/ml) of two different mating types were washed, pre-starved (~12-24 hr) and mixed in 10 mM Tris (pH 7.5) at 30 °C.

Constructions of gene knockout and transgenic strains

To make the *HA-TWI11* construct (Supplementary Figure S1A), an HA coding sequence was inserted immediately after the start codon of *TWI11* by overlapping PCR. The HA sequence, followed by the N-terminal segment of *TWI11*, was amplified with the *TWI11*-FW9-HA and *TWI11*-RV12-Xho primers (Product-A). Part of the 5’ flanking sequences, the 5’-UTR, the start codon, and the HA sequences were amplified by the first PCR using *TWI11*-FW7-Spe + *TWI11*-VR7-EBS (Product-B) and *TWI11*-FW8-EBS + *TWI11*-RV8-HA (Product-C) primers, and overlapping PCR was then used to connect Product-B and Product-C with *TWI11*-FW7-Spe + *TWI11*-RV8-HA (Product-D). This overlapping PCR produced a SmaI site in the middle of the 5’ flanking region. Next, Product-A and

Product-D were joined by overlapping PCR to *TWI11*-FW7-Spe and *TWI11*-RV12-Xho and inserted at the SpeI and XhoI sites of the pBlueScript SK(+) vector. Finally, the *neo3* cassette was introduced into the SmaI site. The construct was excised from the vector backbone using SpeI and XhoI and introduced into conjugating B2086 and CU428 cells by biolistic transformation. Paromomycin-resistant progeny cells were selected, and cells with the *HA-TWI11* locus in the MIC were selected by genomic PCR. Then, *HA-TWI11* loci in the MAC were removed by phenotypic assortment to obtain HA-*TWI11* heterozygous heterokaryon strains. Next, *HA-TWI11* heterozygous heterokaryon strains were mated with the "star strain" B*6 to obtain *HA-TWI11* homozygous heterokaryon (*HA-TWI11* MIC replacement) strains. The replacement of both *TWI11* loci in the MIC with the *HA-TWI11* construct was confirmed by genomic PCR with *TWI11*-MicLC-FW1 and *TWI11*-RV5-BBC.

The *TWI11* KO construct (Supplementary Figure S1I) was made by overlapping PCR. Two parts of the genomic region, including the *TWI11* gene, were amplified by PCR using *TWI11*-FW3-Spe + *TWI11*-VR5-BBC (Product-E) or *TWI11*-FW4-SCA + *TWI11*-RV4-Xho (Product-F). The *neo3* cassette was amplified by PCR using MnB-FW-BBC and MnB-RV-SCA from pNeo3. Next, Product-E, *neo3* and Product-F were joined by overlapping PCR with *TWI11*-FW3-Spe and *TWI11*-RV4-Xho. This product was introduced into conjugating B2086 and CU428 cells through biolistic transformation. Paromomycin-resistant progeny cells were selected, and cells with the *TWI11* KO locus in the MIC were chosen by genomic PCR. Then, *TWI11* KO loci in the MAC were removed by phenotypic assortment to obtain *TWI11* KO heterozygous heterokaryon strains. *TWI11* KO heterozygous heterokaryon strains were then mated with the "star strain" B*6 or

7 to obtain *TWI11* KO homozygous heterokaryon (*TWI11* MIC-KO) strains. The absence of the *TWI11* gene in the MIC was confirmed by genomic PCR with *TWI11*-MicLC-FW1 and TWI11-MicLC-RV1.

The *TWI1* MIC-KO (Mochizuki and Gorovsky, 2004) and *TWI11* MIC-KO strains were mated to obtain *TWI1/TWI11* double KO heterozygous cells. Then, *TWI1* and *TWI11* KO loci in the MAC were removed by phenotypic assortment to obtain *TWI1/TWI11* double KO heterozygous heterokaryon strains. Next, *TWI1/TWI11* double KO heterozygous heterokaryon strains were mated with the "star strain" B*6 or 7 to obtain *TWI1/TWI11* double KO homozygous heterokaryon strains (*TWI1/TWI11* double MIC-KO) strains. The absence of *TWI1* and *TWI11* genes in the MIC was confirmed through genomic PCR with *TWI1*-mic-CasMut-cFW2 and *TWI1*-mic-cRV1 or *TWI11*-MicLC-FW1 and *TWI11*-MicLC-RV1 (Supplementary Figure S1I). The DNA oligos used to make the targeting/transgenic constructs are listed in Supplementary Table 2.

To produce *MTT1*:: FLAG-HA-*TWI1* strains, which express FLAG-HA-*TWI1* from *MTT1* promoter in the MAC, FLAG-HA-*TWI1* coding sequence was amplified by PCR using the previously reported FLAG-HA-*TWI1*-WT construct (Noto et al. 2010) as template and FH-TWI1_pBMMB1_FW and FH-TWI1_pBMMB1_RV as primers. The PCR product was connected to BamHI and SpeI digested pBNMB1 (Busch et al. 2010) by Gibson Assembly kit. The resulted plasmid was the digested with XhoI and introduced into the MAC of the wild-type B2086 and CU428 strains. Phenotypic assortment was performed until the cells grew in 20-30 mg/mL of paromomycin. The strains derived from B2086 and CU428 were mated, refeeded at 3 hpm by adding SPP medium to the final concentration of

0.1x, and expression of FLAG-HA-*TWI1* was induced by adding 0.5 μg/mL CdCl2 at 5 hpm.

Antibodies

To generate the anti-Twi11p antibody, a rabbit was immunized with the fusion protein of MBP and the C-terminal half (250-798 aa) of Twi11p, expressed in *E. coli*. The rabbit polyclonal antibody for Twi1p was described previously (Aronica et al., 2008). The mouse monoclonal anti-HA antibody HA.11 clone 16B12 (Covance) is commercially available. The mouse monoclonal anti-alpha-tubulin antibody 12G10 was obtained from the Developmental Studies Hybridoma Bank at the University of Iowa.

Positions of IESs

The positions of IESs used for the analyses shown in Figure 4 in the draft MIC genome sequence are IES5: SC2.1 104,037-107,601; IES737: SC2.8 130,820-133,697; IES1147 SC2.12 1,551,243-1,553,804; IES1988: SC2.26 393,792-398,354, IES4092: SC2.80 145,860-148,725; and IES4874: SC2.110 112,688-121,910.

IESs, CDSs, TEs and Repeats distributions

The previously predicted IESs (Schoeberl et al., 2012) and manually annotated 28 IESs are listed in Supplementary Table S1. The draft MIC genome sequence (version 2) was obtained from the *Tetrahymena* Comparative Sequencing Project (Broad Institute of Harvard and MIT). The list of predicted gene CDSs (version released in October 2008 by the J. Craig Venter Institute) was obtained from the

Tetrahymena Genome Database (http://ciliate.org/index.php/home). To visualize the distributions of IESs and CDSs, their occupancies in the assembled MIC genome were calculated. Introns were also considered CDSs in this study. As representative transposable elements (TEs), two previously published *Tetrahymena* TE families, the Tlr1 and REP elements (Fillingham et al., 2004; Wuitschick et al., 2002), were used. The following sequences, obtained from GenBank, were compiled as TE sequences: AF451863; AF451864; AF451865; AF451862; AF451860.1; AF232244; F232247.1; AF232243; AF232245; AF232242.1; AF451867; AF451866; AF451869.1; AF451861.1; AF451868.1; AY371728.1; AY371729.1; AY371730.1; AY371731.2. All possible 25-nt sequences were extracted from the compiled TE sequences, and the occupancies of these 25-nt sequences in the assembled MIC genome were calculated by allowing multiple mapping to the genome. To analyze repeat frequencies, the total draft MIC genome, total Type-A IESs and total Type-B IESs were analyzed separately. See below for Type-A and Type-B IES classification. Repeats were analyzed in a manner similar to TEs, although the frequency of 25-nt sequences but not their occupancy was counted.

IES Classification

Among the annotated IESs (listed in Supplementary Table S1), 8,105 IESs longer than 300 nt and containing more than 100-nt A/C/G/T bases were used for the classification. Normalized (RPKM) and weighted (read number of each sequence was divided by the frequencies of the sequence in the draft MIC genome) numbers of small RNA reads mapping to each IES were obtained from small RNAs co-precipitated with HA-Twi11p at 10.5 hpm (=L) and oxidation-resistant

(= Twi1p-bound) small RNAs at 3 hpm (=E). The oxidation-resistant small RNAs at 3 hpm were previously described (Schoerbel et al. 2012). If L of an IES was lower than 1 RPKM, the IES was classified as a Type-C IES. If L of an IES was greater than or equal to 1 RPKM, the Late-scnRNA/Early-scnRNA index (LEI) was calculated as L/E. If E was 0, 0.01 was used for E instead. IESs with LEIs lower than 10 were defined as Type-A IESs. The remaining IESs were classified as Type-B IESs. Type-A and Type-B IESs were further divided into five subclasses: A1 (LEI ≤1); A2 (1< LEI ≤4); A3 (4<LEI ≤10); B1 (10< LEI ≤40); B2 (LEI >40).

Purification of the new MAC and DNA elimination analysis

Nuclei from exconjugants at 36 hpm were dissociated and stained with DAPI in TMSNP buffer (0.25 M sucrose, 10 mM Tris-HCl pH 7.5, 10 mM $MgCl_2$, 3 mM $CaCl_2$, 0.016% NP-40, 1 mM PMSF, 1x complete proteinase inhibitor cocktail (Roche), 0.1 µg/mL DAPI) by a dounce homogenizer with 15 strokes. The nuclear fraction was collected by centrifugation at 4,500 g at 4°C for 5 min, washed with TMSN buffer (TMSNP buffer without PMSF) 3 times, and stored at -80˚C. The nuclear fraction was resuspended with TMSN buffer containing 2 mM EDTA and 0.016 or 0.1% NP-40, and the new MACs were collected by FACSAriaIII (BD biosciences) according to size and DAPI intensity. The purity of the new Mac was assessed by immunostaining using an anti-H3K4me3 antibody (Abcam) and DAPI staining, with H3K4me3-negative nuclei counted as MICs; H3K4me3-positive, DAPI-poor small nuclei counted as new MACs; and H3K4me3-positive, DAPI-rich large nuclei counted as parental (old) MACs. Contamination with the

parental (old) MACs was rare (<1 %), and most of the contaminants in our new MAC preparations were MICs.

DNA elimination analysis with pseudo-IES

The R-IES (MIC supercontig 2.231, 124320-125409, LEI= 10.53) and its flanking sequences were amplified as two (left and right) pieces by PCR from the total genomic DNA of B2086 using the primers RfGFP_R5FW and R_Mic_clone_RV (for left) and R_Mic_clone_FW and RfGFP_R5RV (for right). These two PCR products were then connected by overlapping PCR using the RfGFP_R5FW and RfGFP_R5RV primers. To produce the "pseudo-IES," the left arm of the R-IES (20 bp of R-IES and 224 bp of the left flanking MDS) was amplified by PCR with RfGFP_R5FW and RfGFP_R5RV; the first half of the EGFP-encoding gene (Kataoka et al., 2010) was amplified with RfGFP_GFP5FW and RfGFP_GFPmRV; the second half of the EGFP-encoding gene was amplified with RfGFP_GFPmFW and RfGFP_GFPRV; and the right arm of R-IES (20 bp of R-IES and 288 bp of the right flanking MDS) was amplified by PCR with RfGFP_R5FW and RfGFP_R5RV. Then, the four PCR products were connected by overlapping PCR using the RfGFP_R5FW and RfGFP_R5RV primers. This overlapping PCR produces a NotI site in the middle of the EGFP sequence. Then, the R-IES and the pseudo-IES were digested with PspOMI and cloned into the NotI site of the extra-chromosomal (rDNA) vector pD5H8 (gift from Dr. Meng-Chao Yao, Academia Sinica, Taiwan) to produce pD5H8-R-IES and pD5H8-pseudo-IES.

coDel

M-IES (MIC supercontig 2.231: 120,715-12,1623, LEI=1.06) and its flanking sequences were amplified as two (left and right) pieces by PCR from the total genomic DNA of B2086 using the primers McoDel_5FW_PspOMI and McoDel_IESRV_Not (left) and McoDel_IESFW_Not and McoDel_3RV_PspOMI (right). Then, these two PCR products were connected by overlapping PCR using McoDel_5FW_PspOMI and McoDel_3RV_PspOMI. This overlapping PCR produced a NotI site in the middle of the IES. CL1-IES (MIC supercontig 2.218: 108,618-111,224, LEI=0.82) and CR5-IES (MIC supercontig 2.218: 40,058-46,513, LEI = 0.18) were amplified similarly using CL1coDel_5FW_PspOMI/CL1coDel_IESRV_Not/CL1coDel_IESFW_Not/CL1coDel_3RV_PspOMI and CR5coDel_5FW_PspOMI/CR5coDel_IESRV_Not/CR5coDel_IESFW_Not/CR5coDel_3RV_PspOMI, respectively. The PCR products were digested with PspOMI and cloned into the NotI site of pD5H8 to produce the pMcoDel, pCL1coDel and pCR5coDel vectors. These vectors were digested with NotI, and target sequences were inserted by Gibson Assembly (NEB). The target sequences used in this study were as follows: TTHERM_00079530 (GenBank GG662704: 1,004,795-1,005,509); TTHERM_00841280 (GenBank GG662249: 38,458-39,065); and non-coding sequence on MAC_Contig 3814 (GenBank GG662798: 1,349,433-1,350,093). These target sequences were PCR-amplified with the primer sets cDelSa1_FW/cDelSa1_RV, cDelSa18_FW/cDelSa18_RV, and cDelMC3812_FW/cDelMC3812_RV, respectively. The vectors were introduced into wild-type or *TWI11* MIC-KO cells as described in the section "DNA elimination analysis with pseudo-IES," and transformants were selected with 100 μg/mL paromomycin in 1x SPP. Total genomic DNA was extracted from

paromomycin-resistant cells using a NucleoSpin Tissue Kit (Macherey-Nagel), and DNA eliminations at the endogenous TTHERM_000795, TTHERM_00841280, and non-coding sequence on MAC_Contig 3814 loci were analyzed by PCR using TTHERM_00079530_DelCheck_FW/TTHERM_00079530_DelCheck_RV, Sa18_DelCheck_FW/Sa18_DelCheck_RV, or MC3812_DelCheck_FW/MC3812_DelCheck_RV, respectively. The primers used are listed in Table S2.

Simulation of accidental loss of Early-scnRNA expression and targeting of protein coding sequences

Among the classified Type-A, -B and -C IESs, examples longer than 500 nt and possessing more than 250 nt of A/C/G/T sequences were used as the total set of IESs (7,949 total: 4634 Type-A, 3264 Type-B, and 51 Type-C). A set of 46 (1%) or 463 (10%) non-redundant Type-A IES was randomly chosen, and all possible continuous 20-mers were extracted from the chosen Type-A IESs. Then, the extracted 20-mers were mapped to every IES. IESs containing more than 250 nt to which the 20-mers mapped in at least one of any possible continuous 500-nt segments were identified as recognized IESs (1° IESs). Then, all possible 20-mers were extracted from 1° IESs, and the same calculation was repeated to identify 2° IESs. These simulations were performed using three different sets of randomly chosen Type-A IESs for each condition (1% or 10%), and the means of the fractions of 1° and 2° IESs were calculated.

To analyze the potential targeting of the protein CDS by IES-derived scnRNAs, the predicted CDSs (see above) longer than 500 nt and possessing more than 250 nt of A/C/G/T sequences were used as the total set of CDSs

(20,491 in total). All possible continuous 20-mers were extracted from all of the predicted IESs, and the extracted 20-mers were mapped to every CDS. CDSs containing more than 250 nt to which the 20-mers were mapped in at least one of the possible continuous 500-nt segments were identified as recognized CDSs.

References for Supplemental Experimental Procedures

Aronica, L., Bednenko, J., Noto, T., DeSouza, L.V., Siu, K.W., Loidl, J., Pearlman, R.E., Gorovsky, M.A., and Mochizuki, K. (2008). Study of an RNA helicase implicates small RNA-noncoding RNA interactions in programmed DNA elimination in *Tetrahymena.* Genes & Development *22*, 2228-2241.

Fillingham, J.S., Thing, T.A., Vythilingum, N., Keuroghlian, A., Bruno, D., Golding, G.B., and Pearlman, R.E. (2004). A non-long terminal repeat retrotransposon family is restricted to the germ line micronucleus of the ciliated protozoan *Tetrahymena thermophila*. Eukaryotic Cell *3*, 157-169.

Gorovsky, M.A., Yao, M.C., Keevert, J.B., and Pleger, G.L. (1975). Isolation of micro- and macronuclei of *Tetrahymena pyriformis*. Methods in Cell Biology *9*, 311-327.

Kataoka, K., Schoeberl, U.E., and Mochizuki, K. (2010). Modules for C-terminal epitope tagging of *Tetrahymena* genes. Journal of Microbiological Methods *82*, 342-346.

Mochizuki, K., Fine, N.A., Fujisawa, T., and Gorovsky, M.A. (2002). Analysis of a *piwi*-related gene implicates small RNAs in genome rearrangement in *Tetrahymena*. Cell *110*, 689-699.

Mochizuki, K., and Gorovsky, M.A. (2004). Conjugation-specific small RNAs in *Tetrahymena* have predicted properties of scan (scn) RNAs involved in genome rearrangement. Genes & Development *18*, 2068-2073.

Mochizuki, K., and Gorovsky, M.A. (2005). A Dicer-like protein in *Tetrahymena* has distinct functions in genome rearrangement, chromosome segregation, and meiotic prophase. Genes & Development *19*, 77-89.

Noto, T., Kurth, H.M., Kataoka, K., Aronica, L., DeSouza, L.V., Siu, K.W., Pearlman, R.E., Gorovsky, M.A., and Mochizuki, K. (2010). The *Tetrahymena* argonaute-binding protein Giw1p directs a mature argonaute-siRNA complex to the nucleus. Cell *140*, 692-703.

Schoeberl, U.E., Kurth, H.M., Noto, T., and Mochizuki, K. (2012). Biased transcription and selective degradation of small RNAs shape the pattern of DNA elimination in *Tetrahymena*. Genes & Development *26*, 1729-1742.

Wuitschick, J.D., Gershan, J.A., Lochowicz, A.J., Li, S., and Karrer, K.M. (2002). A novel family of mobile genetic elements is limited to the germline genome in *Tetrahymena thermophila*. Nucleic Acids Research *30*, 2524-2537.
