# Supplementary material for: Small-RNA-Mediated Genome-wide trans-Recognition Network in Tetrahymena DNA Elimination
Source: Mol Cell. 2015 Jul 16;59(2):229–42. doi: 10.1016/j.molcel.2015.05.024 (PMC4518040; doi:10.1016/j.molcel.2015.05.024)
Supplement: Document S2. Article plus Supplemental Information [file mmc4.pdf]

Molecular Cell

Article

Small-RNA-Mediated Genome-wide *trans*-Recognition Network in *Tetrahymena* DNA Elimination

Graphical Abstract

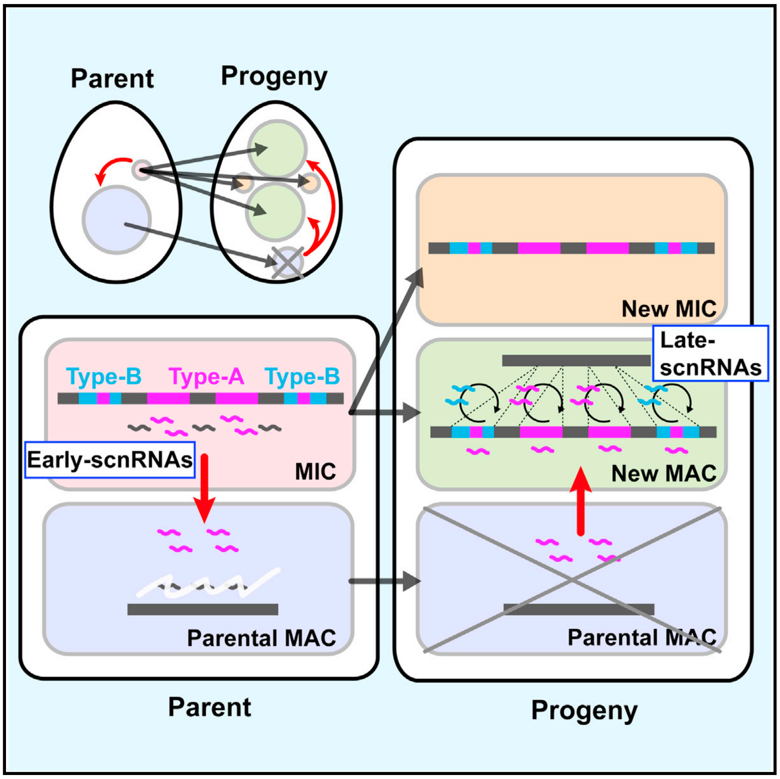

Authors

Tomoko Noto, Kensuke Kataoka,
Jan H. Suhren, ..., Katrina J. Woolcock,
Martin A. Gorovsky,
Kazufumi Mochizuki

Correspondence

kazufumi.mochizuki@imba.oeaw.ac.at

In Brief

Noto et al. show that a class of *Tetrahymena* small RNAs from internal eliminated sequences (IESs) containing potentially active transposons recognize not only the IESs from which they are derived but also other IESs in *trans*, triggering the expression of another class of small RNAs and ensuring robust DNA elimination.

Highlights

- Two types of siRNAs (scnRNAs) are expressed in *Tetrahymena* sexual reproduction
- Early-scnRNAs are produced from Type-A IESs containing potentially active transposons
- Early-scnRNAs *trans*-recognize Type-B IESs and trigger late-scnRNA production in *cis*
- Late-scnRNA production forms *trans*-recognition network for robust IES elimination

Accession Numbers

GSE68455
GSE68457

Noto et al., 2015, Molecular Cell *59*, 229–242
July 16, 2015 ©2015 The Authors
http://dx.doi.org/10.1016/j.molcel.2015.05.024

CellPress

Small-RNA-Mediated Genome-wide *trans*-Recognition Network in *Tetrahymena* DNA Elimination

Tomoko Noto,[1,3] Kensuke Kataoka,[1,3] Jan H. Suhren,[1] Azusa Hayashi,[1] Katrina J. Woolcock,[1] Martin A. Gorovsky,[2] and Kazufumi Mochizuki[1,*]
[1]Institute of Molecular Biotechnology of the Austrian Academy of Sciences (IMBA), Dr. Bohr-Gasse 3, 1030 Vienna, Austria
[2]Department of Biology, University of Rochester, Rochester, NY 14627, USA
[3]Co-first author
*Correspondence: kazufumi.mochizuki@imba.oeaw.ac.at
http://dx.doi.org/10.1016/j.molcel.2015.05.024
This is an open access article under the CC BY license (http://creativecommons.org/licenses/by/4.0/).

SUMMARY

Small RNAs are used to silence transposable elements (TEs) in many eukaryotes, which use diverse evolutionary solutions to identify TEs. In ciliated protozoans, small-RNA-mediated comparison of the germline and somatic genomes underlies identification of TE-related sequences, which are then eliminated from the soma. Here, we describe an additional mechanism of small-RNA-mediated identification of TE-related sequences in the ciliate *Tetrahymena*. We show that a limited set of internal eliminated sequences (IESs) containing potentially active TEs produces a class of small RNAs that recognize not only the IESs from which they are derived, but also other IESs in *trans*. This *trans* recognition triggers the expression of yet another class of small RNAs that identify other IESs. Therefore, TE-related sequences in *Tetrahymena* are robustly targeted for elimination by a genome-wide *trans*-recognition network accompanied by a chain reaction of small RNA production.

INTRODUCTION

Transposable elements (TEs) are threats to host genome integrity in addition to being drivers of host genome evolution. Hosts have therefore developed defense mechanisms to convert TEs into silent residents (Levin and Moran, 2011). Different eukaryotes use a variety of RNAi-related mechanisms to distinguish TEs from the host genome and to downregulate them. In mammals and flies, TE silencing is mediated by Piwi-interacting RNAs (piRNAs) that are produced from genomic loci, called piRNA clusters, where accumulation of TE remnants creating a genetic memory of TEs (Guzzardo et al., 2013; Siomi et al., 2011). In nematodes, however, piRNAs have enormous targeting capacity beyond TEs and are counteracted by CSR-1-bound 22G-RNAs that hold an epigenetic memory of previous gene expression (Seth et al., 2013; Wedeles et al., 2013). In contrast to these memory-based TE identification systems in metazoans, plants and yeasts utilize intrinsic features of TEs for their identification. In *Arabidopsis* pollen formation, the active de-silencing of TEs in somatic cells causes accumulation of TE-derived aberrant RNAs, producing small interfering RNAs (siRNAs), which then move into an adjacent germline cell to reinforce TE silencing (Slotkin et al., 2009). The yeasts *Cryptococcus neoformans* and *Schizosaccharomyces pombe* use transcripts with suboptimal RNA structure in the host-splicing machinery as a sign of non-self genetic material and compose siRNAs from these transcripts to silence TEs (Dumesic et al., 2013; Lee et al., 2013).

Nuclear dimorphism allows ciliated protozoans to identify TEs in a unique way (Chalker et al., 2013). Most ciliates contain two types of nuclei in a single cell: the transcriptionally silent germline micronucleus (MIC) and the transcriptionally active somatic macronucleus (MAC). During sexual reproduction (conjugation, Figure 1A), the MIC produces new MICs and MACs, while the parental MAC is discarded. TEs and their remnants are mostly removed from the new MAC by programmed DNA elimination (Arnaiz et al., 2012; Chen et al., 2014; Coyne et al., 2012). In the two closely related oligohymenophorean ciliates *Paramecium* and *Tetrahymena*, genome-wide production of siRNAs (called scnRNAs) occurs in the MIC upon sexual reproduction. These scnRNAs then move into the parental MAC, where scnRNAs complementary to its genome are degraded. The remaining MIC-specific scnRNAs are then transferred into the new MAC, where they target complementary DNA for elimination (Mochizuki et al., 2002; Sandoval et al., 2014; Schoeberl et al., 2012). In a conceptually similar strategy, the more distantly related spirotrichous ciliates (e.g., *Oxytricha*) produce small RNAs from the parental MAC that then protect their complementary DNA in the new MAC from elimination (Fang et al., 2012). Both mechanisms identify TEs and their remnants as sequences present in the MIC, but not in the MAC.

In *Tetrahymena*, DNA elimination reproducibly removes >8,000 DNA segments, called internal eliminated sequences (IESs), that comprise ∼30% of the MIC genome (Coyne et al., 2012). IESs include all known TEs and certain unique sequences that are potentially remnants of TEs (Eisen et al., 2006; Yao et al., 2003). *Tetrahymena* scnRNAs (∼26- to 32-nt [mainly 28- to 30-nt] siRNAs) are produced in the MIC by the Dicer protein Dcl1p (Malone et al., 2005; Mochizuki and Gorovsky, 2005) and are loaded into the Argonaute protein Twi1p (Noto et al., 2010; Woehrer et al., 2015). The absence of Dcl1p or Twi1p completely blocks DNA elimination, indicating that scnRNAs are necessary for the elimination of all IESs.

CrossMark

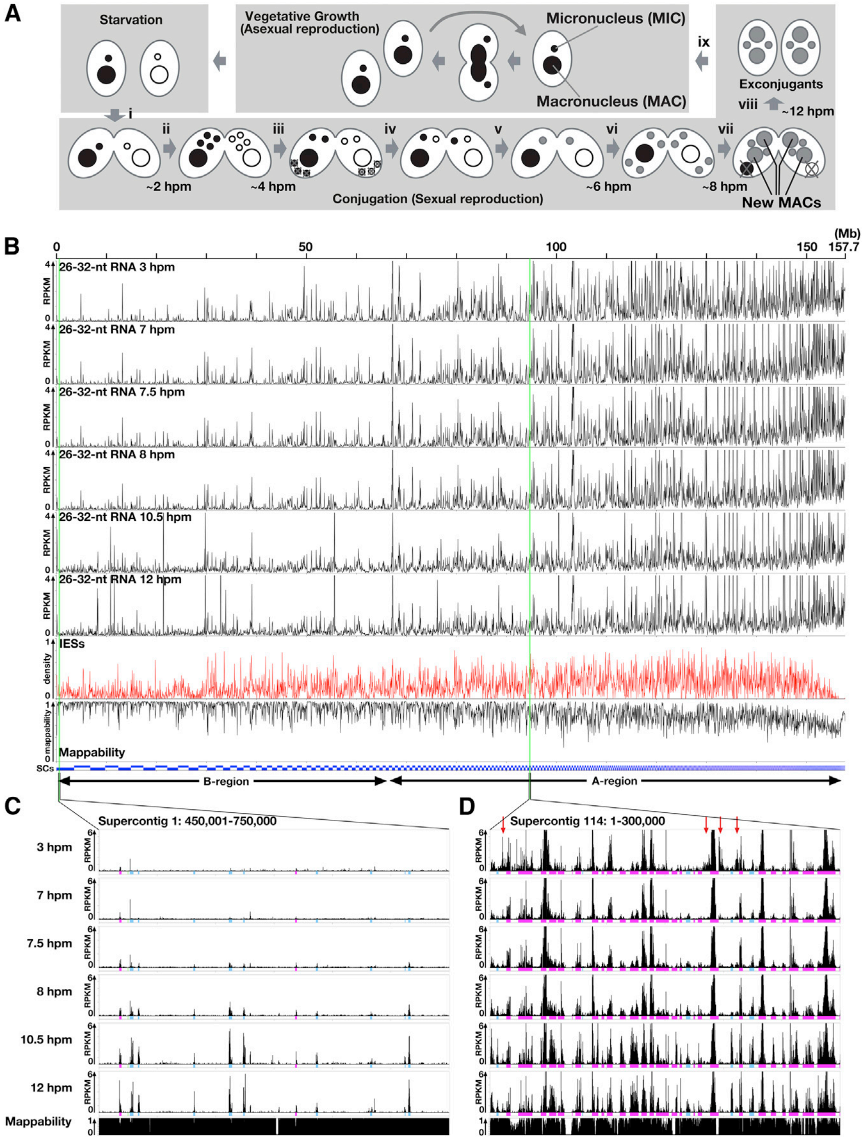

Figure 1. Two Types of scnRNAs

(A) A *Tetrahymena* cell contains a macronucleus (MAC) and a micronucleus (MIC). During vegetative growth, both the MAC and the MIC divide and segregate to daughter cells. Mixing starved cells of different mating types induces conjugation (i). The MICs undergo meiosis (ii), and one of the selected products divides mitotically to form two pronuclei (iii). One of the pronuclei crosses the conjugation bridge (iv) and fuses with the stationary pronucleus to produce the zygotic nucleus (v), which then divides twice (vi) to form two new MACs and two MICs (vii). The parental MAC is degraded, and the pair is dissolved (viii). The exconjugants resume vegetative growth when the nutrient supply is restored (ix). The approximate time when each event occurs is indicated (hpm, hours post-mixing).

(B) 1,464 MIC genome supercontigs (SCs, blue bars) were ordered by their lengths (longest to shortest) and concatenated. Normalized numbers (reads per kilobase per million reads [RPKM]) of sequenced 26- to 32-nt RNAs from WT cells at the indicated time points that map uniquely to the MIC genome are shown as histograms with 50-kb bins. The densities of IESs and mappable (unique) sequences are also shown. The drop in IES density in the region containing very short SCs is probably because these SCs are shorter than most of the IESs, and the prediction of IESs from them failed. The regions enlarged in (C and D) are marked with green lines. Longer (1–50) and shorter (51–1,464) MIC SCs represent B- and A-regions of the MIC genome, respectively.

(C and D) Small RNA expression from the indicated 300-kb windows (shown and analyzed as in B, except with 100-nt bins). Colored boxes indicate the positions of IESs (magenta, type A; sky blue, type B; see Figure 3 for the IES classification). In (D), the arrows mark MAC-destined regions that are the origins of Early-scnRNAs that accumulated prominently at early stages (3 hpm) but were degraded later.

It has been suggested that IESs in *Tetrahymena* are identified solely by scnRNA-mediated subtraction of the MAC genome sequence from the MIC genome (Mochizuki et al., 2002). However, in such a genome-subtraction system, any errors in DNA elimination can be transgenerationally inherited. Therefore, there could be additional mechanisms ensuring the accuracy of DNA elimination. Furthermore, our previous report that the production of scnRNAs from the MIC genome is non-uniform (Schoeberl et al., 2012) indicates that an additional mechanism beyond simple genome-subtraction is involved in DNA elimination. In this study, we describe a small RNA-mediated regulation of DNA elimination in *Tetrahymena*, in which a genome-wide trans-recognition network functions in a piRNA pathway-like memory-based genome surveillance and information amplification system to ensure the robust transgenerational transmission of TE memory.

RESULTS

Late-scnRNAs Are Expressed at Late Conjugation Stages

We previously reported that scnRNAs in *Tetrahymena* are produced non-uniformly from the MIC genome at two levels (Schoeberl et al., 2012): (1) although scnRNAs are produced from a variety of regions in the MIC genome, many >100-kb stretches of MIC regions are scnRNA deserts (referred to as global bias); and (2) within scnRNA-producing regions, scnRNAs are preferentially produced from IESs (referred to as local bias). We confirmed these observations by analyzing the production of scnRNAs throughout the MIC genome (Figure 1B). Currently available sequences of the MIC genome, which were derived from five MIC chromosomes, were assembled into 1,464 supercontigs (SCs). These SCs were ordered by length (longest to

shortest; see Figure 1B, "SCs") and concatenated as 158 Mb of linear DNA, which was used as the MIC genome for this study. The first 25 nt of scnRNA sequences (26–32 nt) from wild-type (WT) cells were then mapped to the MIC genome, and the number of scnRNAs mapping uniquely to the MIC genome was determined.

As shown in Figures 1B–1D, at early- (3 hr post-mixing [hpm]) to mid-conjugation (7 hpm), scnRNAs were mainly produced from regions of the MIC genome represented by shorter SCs (for example, SC114; Figure 1D), which are referred to as A-regions. In contrast, scnRNAs were rarely produced from regions represented by longer SCs (for example, SC1; Figure 1C), which are referred to as B-regions. This pattern is partially caused by the local bias because IESs are concentrated in A-regions (Figure 1B, IESs). However, local bias does not fully explain the scnRNA production pattern; although IESs are also present in B-regions, they are not the origin of scnRNAs (Figure 1C, 3 hpm and 7 hpm, where magenta and sky-blue boxes represent IESs). Therefore, we conclude that the global bias is due to a lack of scnRNA production from the B-regions of MIC chromosomes.

In contrast to early conjugation, 26- to 32-nt small RNAs were expressed from B-regions at late conjugation stages (7.5–12 hpm, Figures 1B and 1C) and were produced almost exclusively from IESs (Figure 1C). We refer to small RNAs expressed only at late conjugation stages as "Late-scnRNAs" and scnRNAs expressed in the MIC during early conjugation as "Early-scnRNAs." Henceforth, the generic term scnRNA includes both Early- and Late-scnRNAs. Because DNA-dependent RNA polymerase proteins were detected only in the parental and new MACs at late conjugation (Mochizuki and Gorovsky, 2004) and the parental MACs lack IESs, Late-scnRNAs must be expressed in the new MAC. Late-scnRNAs from most of the B-region IESs were detected as early as 7.5 hpm, immediately after the formation of the new MACs (see Figure 1A) and before the earliest detectable IES excisions (10 hpm) (Austerberry et al., 1984; Saveliev and Cox, 1994), indicating that Late-scnRNAs are produced before the IESs from which they are derived are excised from new MAC chromosomes.

Similar IES-derived siRNAs (iesRNAs) are expressed in *Paramecium* during late stages of sexual reproduction (Sandoval et al., 2014). However, iesRNAs are distinct from *Tetrahymena* Late-scnRNAs in several respects: (1) Late-scnRNAs are produced from IESs prior to their excision, but *Paramecium* iesRNAs are made from excised IESs; (2) some IESs express only Late-scnRNAs in *Tetrahymena,* but most, if not all, *Paramecium* IESs express both scnRNAs (probably the equivalent of Early-scnRNAs) and iesRNAs; (3) Early- and Late-scnRNAs are probably produced by the same Dicer enzyme (see below), but *Paramecium* scnRNAs and iesRNAs are processed by distinct Dicers. The potential functional relevance of these IES-derived small RNAs will be discussed in the Discussion section.

Late-scnRNAs Are Loaded into Two Argonautes

Three Argonaute-encoding genes, *TWI1*, *TWI2*, and *TWI11*, are highly expressed at late conjugation stages (Figure 2A). To identify the Argonaute protein(s) loaded by Late-scnRNAs, we focused on Twi1p and Twi11p, because Twi2p is known to interact with another class of (23- to 24-nt) siRNAs (Couvillion et al., 2009). Consistent with the mRNA expression patterns, Twi1p is accumulated throughout conjugation, whereas Twi11p is only expressed during the late conjugation stages (Figure 2B).

Small RNAs co-precipitated with Twi1p by an anti-Twi1p antibody at 10.5 hpm were analyzed. A fraction of Twi1p-bound 26- to 32-nt small RNAs were derived from B-regions (Figure 2G), indicating that maternal and/or zygotic Twi1p binds to Late-scnRNAs. Next, to distinguish maternal from zygotic Twi1p, FLAG-HA-tagged Twi1p (FLAG-HA-Twi1p) was expressed from a transgene inserted only into the maternal MAC in place of the endogenous *TWI1* gene (MAC replacement, see Figure 2C). The accumulation of maternally expressed FLAG-HA-Twi1p peaked at 2–6 hpm and persisted until 12 hpm (Figure 2D). In contrast, total Twi1p detected by an anti-Twi1p antibody accumulated stably until 14 hpm in the WT cells (Figure 2B). These results indicate that both maternal and zygotic Twi1p are accumulated at late conjugation stages (8–12 hpm); however, B-region-derived 26- to 32-nt small RNAs did not co-precipitate with FLAG-HA-Twi1p at 10.5 hpm (Figure 2H). We suggest that this result was not caused by inhibition of Late-scnRNA loading by the FLAG-HA tag, because we found that B-region-derived 26- to 32-nt small RNAs were co-precipitated with FLAG-HA-Twi1p at 10.5 hpm when FLAG-HA-Twi1p was ectopically expressed by the inducible *MTT1* promoter from 5 hpm (Figure S1L). Therefore, we conclude that maternal Twi1p binds only to Early-scnRNAs, whereas zygotic Twi1p interacts with Late-scnRNAs.

To analyze Twi11p-associated small RNAs, the *TWI11* locus in the MIC was replaced with an HA-*TWI11* gene (Figures S1A and S1B), resulting in zygotic expression of HA-Twi11p from the new MAC at late conjugation (MIC replacement; see Figure 2C). The temporal expression patterns of HA-Twi11p and endogenous Twi11p in WT cells were indistinguishable (compare Figure 2B, α-Twi11p and Figure 2E, α-HA), indicating that Twi11p is mainly zygotically expressed. Small RNAs that co-precipitated with HA-Twi11p shared a length distribution (Figures 2F and S1C) and base composition, including the Dicer-product signature (5′ U bias and A bias for the third base from the 3′ end) (Mochizuki and Kurth, 2013), with Twi1p-bound Early-scnRNAs (Figures S1D–S1G). Therefore, they were most likely produced by the same Dicer, Dcl1p. Twi11p-bound small RNAs were resistant to periodate oxidation (Figure S1H), indicating that, similar to Early-scnRNAs (Kurth and Mochizuki, 2009), they were modified at their 3′ ends, presumably by 2′-O-methylation.

The origins of HA-Twi11p-bound small RNAs included IESs in the B-regions (Figure 2I), indicating that Twi11p binds to Late-scnRNAs. Twi11p may predominantly interact with Late-scnRNAs, although we cannot exclude the possibility that certain Early-scnRNAs dissociate from Twi1p and are reloaded into Twi11p. HA-Twi11p-bound small RNAs mapped relatively homogeneously to the entire MIC genome (Figure 2I, left). This result is in clear contrast to the total (Figure 1B) and Twi1p-bound (Figure 2G, left) scnRNAs at the same stage (10.5 hpm), which mainly mapped to A-regions of the MIC genome. Because the density of IESs is higher in A-regions than B-regions, the homogeneous distribution of the origins of Twi11p-bound small RNAs

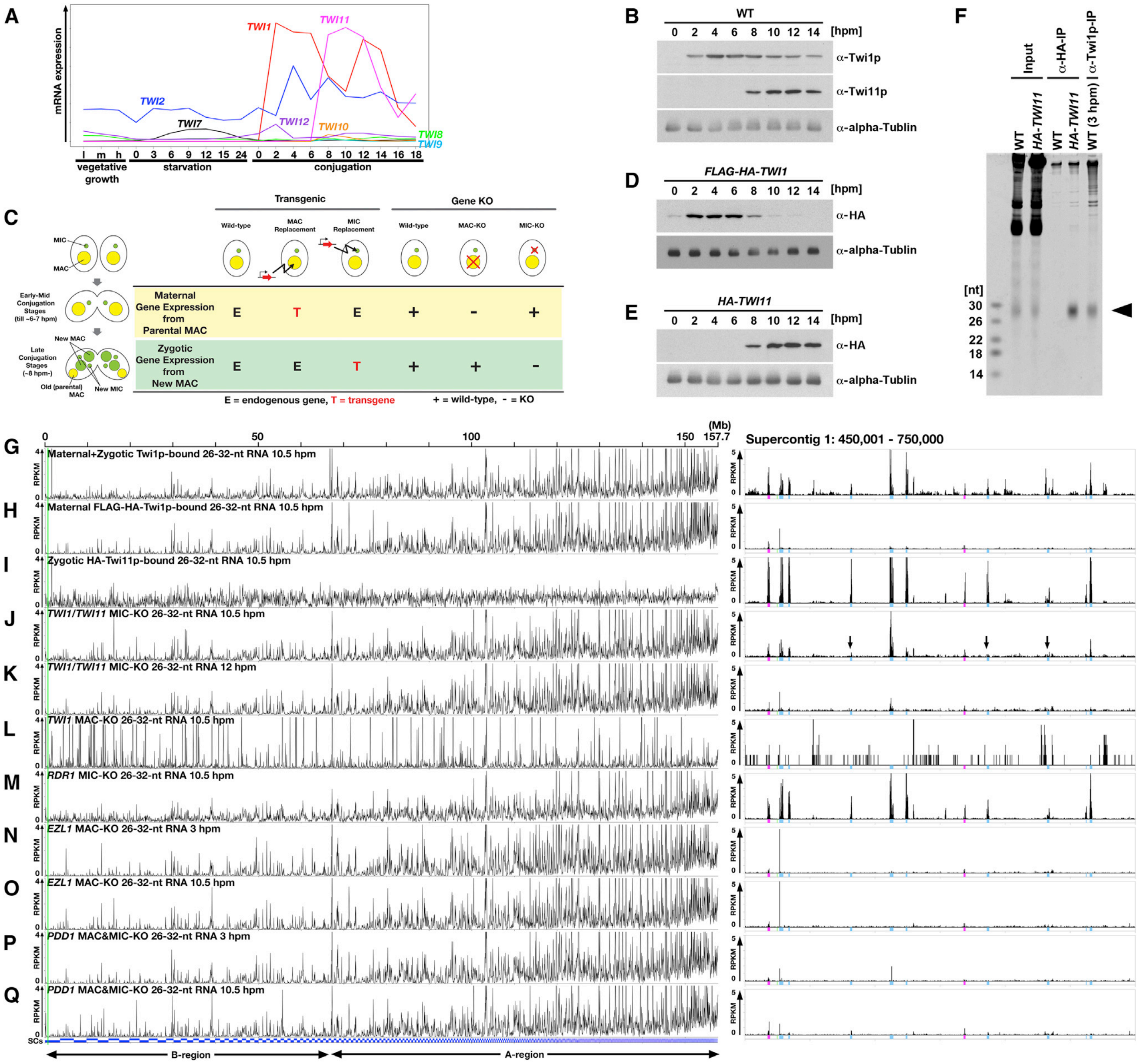

Figure 2. Late-scnRNAs Interact with Twi1p and Twi11p

(A) Expression of the Argonaute genes in vegetatively growing (l, m, and h indicate low, medium, and high density, respectively), starved (numbers indicate hours after removal of nutrients), and conjugating (numbers indicate hpm) cells based on a microarray analysis.

(B) Twi1p and Twi11p from wild-type cells at the indicated time points of conjugation were detected by western blotting using the indicated antibodies.

(C) Different transgenic and gene-knockout strategies. In the vegetative and early- to mid-stage conjugating cells, the parental MACs (yellow) contribute all (maternal) gene expression, whereas the new MACs, which are formed from the MIC (green), provide zygotic gene expression at late conjugation.

(D and E) Proteins from cells expressing FLAG-HA-Twi1p from the maternal *TWI1* loci by MAC replacement (D) or cells expressing HA-Twi11p from the zygotic *TWI11* loci by MIC replacement (E) at the indicated time points of conjugation were detected by western blotting.

(F) HA-Twi11p was immunopurified using an anti-HA antibody (α-HA-IP) from the *HA-TWI11* conjugating culture at 10.5 hpm. As a negative control, wild-type cells (WT) were used for similar immunopurification. Early-scnRNAs were immunopurified with Twi1p by an anti-Twi1p antibody (α-Twi1p-IP) from the wild-type conjugating culture at 3 hpm. RNAs were separated on denaturing gels and stained using a nucleic-acid-specific dye. Arrowhead indicates scnRNAs.

(G–Q) 26- to 32-nt RNAs from the indicated immunopurified RNAs or strains were analyzed as in Figure 1B. The 300-kb window on the right is marked with a green line.

See also Figure S1.

indicates that Late-scnRNAs are preferentially produced from IESs in B-regions.

To understand the importance of Twi1p and Twi11p in the accumulation of Late-scnRNAs, we produced *TWI1* and *TWI11* double-MIC-knockout cells (*TWI1*/*TWI11* MIC-KO), in which both copies of *TWI1* and *TWI11* in the MIC were replaced with a drug-resistance gene (Figures S1I and S1J). A MIC-KO disrupts the zygotic expression of a gene from the new MAC without eliminating its maternal gene expression from the parental MAC (see Figure 2C, MIC-KO). In *TWI1*/*TWI11* MIC-KO cells, Late-scnRNAs from B-region IESs initially accumulated (Figure 2J) but were then largely lost by 12 hpm (Figure 2K), indicating that zygotically expressed Twi1p and Twi11p are dispensable for the biogenesis of Late-scnRNAs but are necessary for their stable accumulation. Therefore, we conclude that Late-scnRNAs are mainly loaded into the zygotically expressed Twi1p and Twi11p.

scnRNA Expression Defines Three Types of IESs

The results above indicate that there are two types of IESs: one that produces Early-scnRNAs and another that only produces Late-scnRNAs. To rigorously classify IESs by these criteria, we calculated Late-scnRNA/Early-scnRNA indexes (LEIs) as ratios of normalized, mapped Twi11p-bound scnRNAs at 10.5 hpm (representing Late-scnRNAs) to mapped Twi1p-bound scnRNAs at 3 hpm (representing Early-scnRNAs) (Figure 3A). Among the 8,105 IESs to which we can reliably map scnRNA data, 4,695 IESs showed LEI $\leq$ 10 (i.e., both types of scnRNAs were expressed) and were defined as Type-A IESs. Type-A IESs were subdivided into three groups: A1 (LEI $\leq$ 1), A2 (1 < LEI $\leq$ 4), and A3 (LEI > 4). The other 3,293 IESs showed LEI > 10 (i.e., Late-scnRNAs were predominantly expressed) and were defined as Type-B IESs. Type-B IESs were subdivided into two groups: B1 (LEI $\leq$ 40) and B2 (LEI > 40). The remaining 117 IESs, which we call type-C IESs, showed very little Twi11p-bound scnRNA expression: <1 reads mapped per kilobase per million reads (RPKM).

LEIs were found to be correlated with the chromosomal locations, lengths, and GC contents of IESs: the lower the LEI of an IES group is, the greater its preference for A-regions (Figure 3B), the longer its mean length (Figure 3C), and the higher its GC contents (Figure 3D). These correlations are likely associated with the "age" of the IESs. We analyzed the localization of sequences related to Tlr1 DNA transposons and REP family retrotransposons (Fillingham et al., 2004; Wuitschick et al., 2002), the known potentially active TE families in *Tetrahymena*. All possible 25-mers were extracted from the previously reported DNA sequences of Tlr1- and REP-family TEs, and their occurrences in the MIC genome were calculated. We found that 98% of the sequences complementary to these two TEs are found in IESs (Figure 3E, left, TEs), which occupy only 23% of the MIC genome sequence we used for the analysis (Figure 3E, left, MIC genome). Within the IESs, the TE-derived sequences are mostly (99.5%) in Type-A IESs (Figure 3E right, TEs), which represent 77% of IESs (Figure 3E, right, IESs). Moreover, these TE-derived sequences co-localize with Type-A1/A2 IESs in the MIC genome (Figure 3B, TEs). Active TE-containing (i.e., young) IESs might have been constrained by natural selection to localize at A-regions, where the production of Early-scnRNAs (Figures 1B and 1D) ensures their DNA elimination from the new MAC. As TE sequences degenerate (i.e., become older), IESs may become shorter, more "tetrahymenanized" by reducing their GC contents in the AT-rich *Tetrahymena* genome (Figure 3D) and tolerated as residents of gene-rich B-regions (see CDSs in Figure 3B), where their DNA elimination occurs independently of their own Early-scnRNA expression (see below).

Late-scnRNAs Are Important for Elimination of Some IESs

To understand the importance of Early- and Late-scnRNAs in eliminating the different types of IESs, new MACs of exconjugants were isolated from different mutants at 36 hpm, and the genomic DNA was analyzed by high-throughput sequencing. As measure for DNA elimination, a retention index (RI; Figure 3F) was calculated for each IES by dividing normalized read numbers (RPM) mapping to an IES by the ones mapping to its flanking MAC-destined sequences. Although in theory, all IESs should show RI = 0 in WT cells, the indexes were typically 0.01-0.1 (Figure 3G), probably due to MIC contamination (~2%–10%) in our new MAC preparations. The variability of RIs between IESs might be due to different endo-replication levels of different MAC chromosomes.

In *TWI1*/*TWI11* MIC-KO cells, in which Late-scnRNAs are unstable (Figures 2J and 2I), RIs of some of Type-B IESs were ~1 (not eliminated), whereas those of most Type-A IESs were ~0.1 (eliminated) (Figure 3H). This result was in sharp contrast to *TWI1* MAC-KO cells, in which both Early- and Late-scnRNAs were lost (Figure 2L), and the RIs of most IESs were ~1 regardless of type (Figure 3I). These findings suggest that Early-scnRNAs are sufficient to induce DNA elimination for a majority of IESs, whereas Late-scnRNAs are important for DNA elimination of some, mainly Type-B, IESs. Elimination of type-C IESs, which lack their own scnRNA production (Figure 3A), was also affected in *TWI1* MAC-KO cells (Figure 3I) and, to a lesser extent, in *TWI1*/*TWI11* MIC-KO cells (Figure 3H), indicating that scnRNAs also play a role in the elimination of Type-C IESs.

trans-Recognition network for IES identification

The sufficiency of Early-scnRNAs for the elimination of many Type-B IESs was puzzling, because Early-scnRNAs from WT cells at 3 hpm rarely map to any Type-B IESs (Figures 1C and 4A–4C, unique mappers). However, in all of the above scnRNA analyses (except for the IES classification in Figure 3A), we only counted sequence reads uniquely mapping to the MIC genome. If we instead allow multiple mapping and normalize for the effect of sequence repetition in the MIC genome (weighted mapping), more Early-scnRNAs mapped to the Type-B IESs (Figures 4A–4C, weighted, marked with arrows), because these Type-B IESs contain some repetitive sequences (Figures 4A–4C, repeats, gray lines) that mostly originate from Type-A IESs (magenta lines). We have termed these repetitive sequences "A-repeats." Although a small fraction of Type-B IESs lack detectable A-repeats (Figure S2), some repetitive sequences sufficient for recognition by Early-scnRNAs might be overlooked because we only considered perfectly complementary 25-nt stretches to identify repetitive sequences.

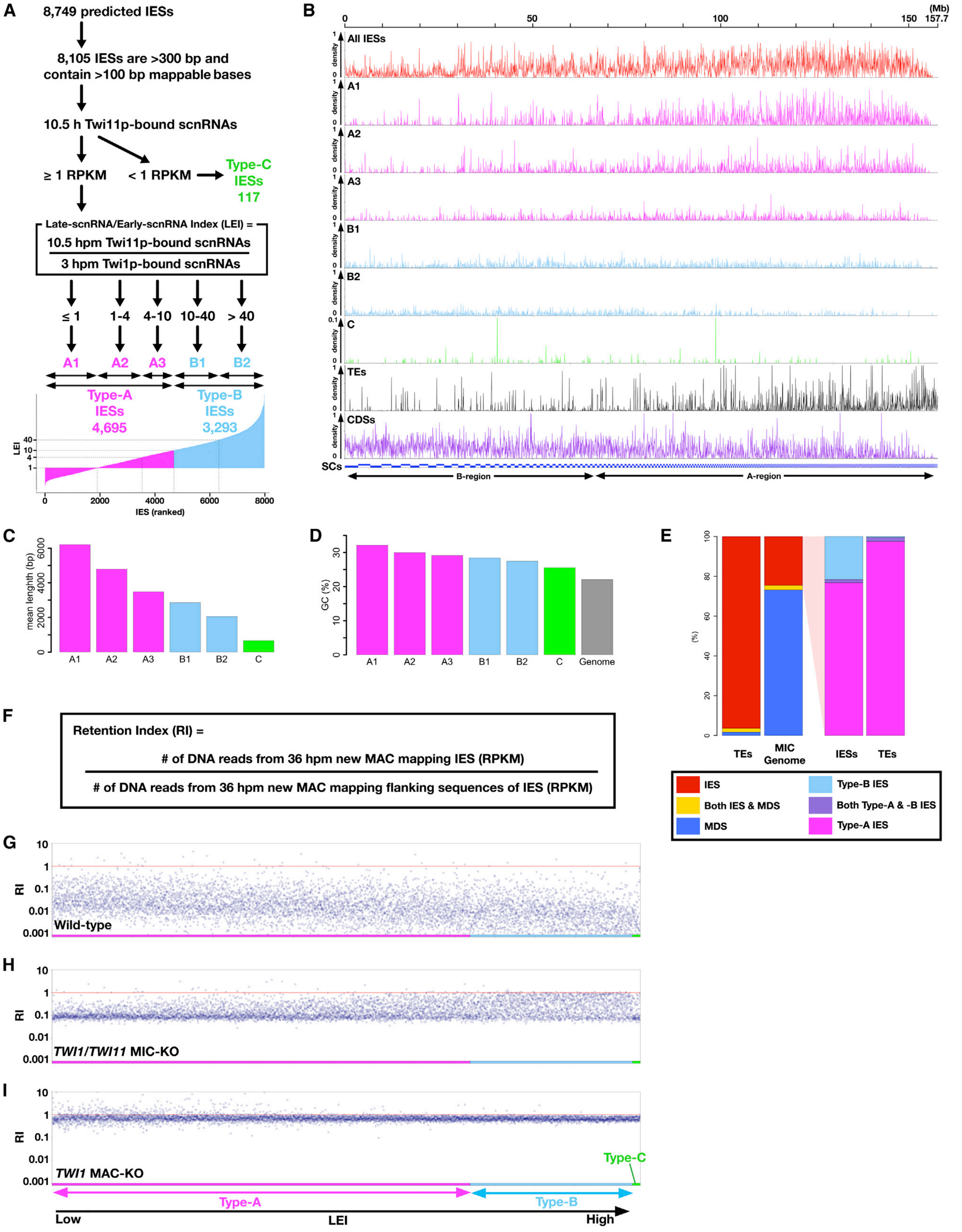

(legend on next page)

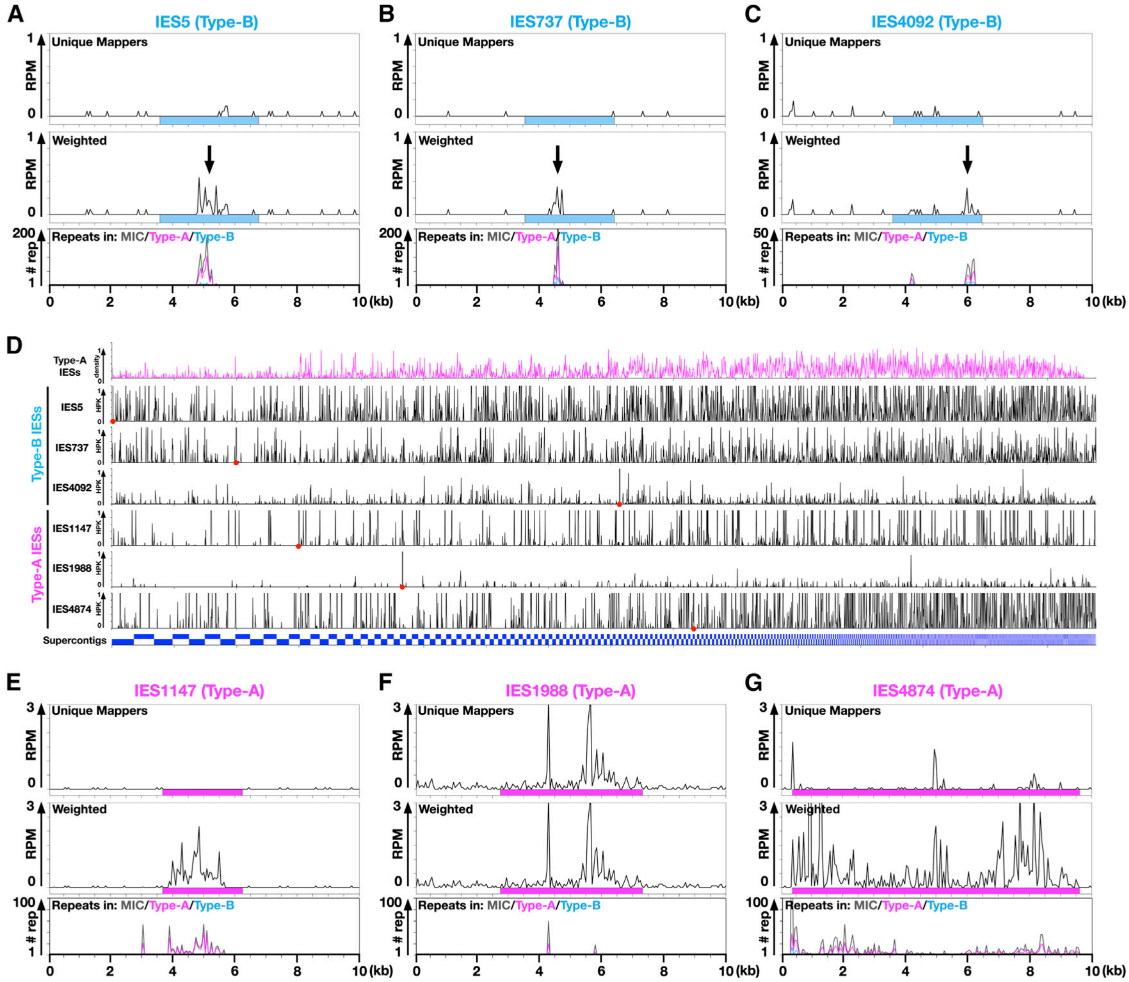

Figure 4. *trans* Recognition of IESs

(A–C) Top two panels: normalized numbers (RPM) of sequenced 26- to 32-nt RNAs from wild-type cells at 3 hpm mapping to the three representative Type-B IES loci (10 kb) are shown as histograms with 50-nt bins. For the top histograms, only the numbers of sequences uniquely mapping to the MIC genome are shown (unique mappers). The middle histograms show the numbers of sequence reads mapping to each position within the loci divided by the total numbers of sites in the entire MIC genome to which the sequence reads map (weighted). Arrows indicate regions to which Early-scnRNAs map. Sky-blue boxes represent the IESs. Bottom panels (repeats): all possible 25-mers from the entire MIC genome sequence (gray), from Type-A IESs (magenta), or from Type-B IESs (sky blue) were mapped to the three Type-B IES regions, and their frequencies of occurrence are shown as histograms with 50-nt bins.

(D) All possible 25-mers were extracted from the indicated IESs, and their frequencies of occurrence (hits per kilobase [HPK]) on the MIC genome are shown as histograms with 50-kb bins. The locations of the IESs are marked with red dots. The density of the Type-A IESs is shown at the top.

(E–G) Three representative Type-A IES (magenta boxes) loci were analyzed as in (A)–(C).

See also Figure S2.

To understand the origins of A-repeats in Type-B IESs, all possible 25-mers were extracted from each Type-B IES, and their occurrence in the MIC genome was analyzed. We found that, for all analyzed Type-B IESs, sequences complementary to each Type-B IES were distributed throughout the MIC genome (Figure 4D), indicating that A-repeats in a Type-B IES are

Figure 3. Three Types of IESs

(A) Classification of IESs according to the expression of Early- and Late-scnRNAs.

(B) Localization of different types of IESs, transposons (TEs), and coding sequences (CDSs) in the MIC genome are shown in a histogram with 50-kb bins.

(C and D) Mean lengths (C) and GC contents (D) of IESs in different IES classes.

(E) Distributions of TE-related sequences among MIC genome components (left) and IES types (right). All possible 25-mers from the MIC genome sequences were classified as sequences that were complementary to only IESs (red), only MAC-destined sequences (MDSs, blue), or both (yellow). All possible 25-mers from the total IESs were classified as sequences that were complementary to only Type-A IESs (magenta), only Type-B IESs (sky blue), or both (purple). The fraction of TE-derived 25-nt sequences complementary to these DNA classes was calculated.

(F–I) Analysis of DNA elimination efficiency. The retention indexes (RIs) of individual IESs in the purified new MACs of the indicated strains at 36 hpm were plotted. IESs are ordered according to their LEIs (on the x axis). The red lines indicate RI = 1 (no DNA elimination).

complementary to many different Type-A IESs in the MIC genome. These observations suggest that Early-scnRNAs from multiple Type-A IESs could target a Type-B IES for DNA elimination through A-repeats. Because most Type-A IESs also share certain A-repeats with other Type-A IESs (Figures 4D–4G), they may also be identified by Early-scnRNAs in *trans*.

A-Repeats Are Important for Type-B IES Elimination

The importance of A-repeats for Type-B IES elimination was analyzed by an artificial DNA elimination assay (Figure 5). First, the previously characterized R-IES (a Type-B IES), including its flanking sequences, was inserted into an extra-chromosomal vector. As previously reported (Godiska and Yao, 1990), this IES was frequently removed from the vector, similar to its endogenous counterpart, upon introduction into the developing MAC (Figure 5A), although elimination did not occur in all progeny cell lines in this artificial assay. Next, most of the R-IES in the vector was replaced by a GFP-encoding sequence to generate a "pseudo-IES" that could not be eliminated (Figure 5B). Fragments of interest within IESs (red numbered boxes in Figure 5C, left bottom) were then inserted into the middle of the pseudo-IES (Figure 5C, left top). Among the fragments from the two representative Type-B IESs (IES5 and IES4092), we found that only DNA fragments containing A-repeats (fragments 1, 2, and 6 in Figure 5C) had strong activity in restoring DNA elimination of the pseudo-IES, whereas most of the fragments of a Type-A IES (IES1988) enabled the pseudo-IES to be eliminated (fragments 7–9 in Figure 5C). Some restoration of elimination by Type-B IES fragments without A-repeats might be due to "2° recognition" by Late-scnRNAs produced by the endogenous counterparts of the Type-B IESs (see below). Restored DNA elimination mostly did not share boundaries with the original R-IES (Figure 5C), indicating that the elimination boundaries are determined not only by information near the normal IES boundaries but also by the internal IES sequence. Nonetheless, all of the results above indicate that most, if not all, Type-B IESs possess A-repeats through which they can be targeted for DNA elimination in *trans* by Type-A IES-derived Early-scnRNAs.

cis Spreading of Late-scnRNA Production

Further examination of Late-scnRNAs revealed that they are produced from Type-B IESs outside the A-repeats (Figures 6A–6C, 7.5–10.5 hpm), suggesting that Early-scnRNAs recognize A-repeats in *trans*, which could trigger the expression of Late-scnRNAs from *cis* regions adjacent to A-repeats. Consistent with the idea of Early-scnRNA-triggered Late-scnRNA expression, *TWI1* MAC-KO cells, which lack Early-scnRNAs because of the loss of maternally expressed Twi1p (Mochizuki et al., 2002), also failed to express Late-scnRNAs (Figure 2L).

To validate the *cis* triggering of Late-scnRNA production by Early-scnRNAs, we designed another artificial DNA elimination assay (Figure 6D). A Type-A IES (Figure 6, magenta box) was inserted into an extra-chromosomal vector (pD5H8), and a non-IES target sequence (Figure 6, black box) was inserted into the middle of the IES. This chimeric construct was then introduced into the developing MAC. We hypothesized (Figure 6E) that Early-scnRNAs produced from the endogenous Type-A IES in the MIC (i) recognize the Type-A IES on the vector in the new MAC (ii) and trigger Late-scnRNA production from the adjacent target sequence in *cis* (iii), which then recognize the endogenous target locus in *trans* (iv) and induce ectopic DNA elimination (vi).

The introduction of a chimeric construct containing the well-studied M-IES (a Type-A IES) and part of a coding gene efficiently induced ectopic DNA elimination in *trans* at the endogenous chromosomal locus of the gene (Figure 6F), whereas the IES alone (Figure 6G) or the target DNA alone (Figure 6H) in the vector did not. Analogous ectopic DNA elimination was induced with combinations of different Type-A IESs (Figures 6I and 6J) and different coding (Figure 6K) as well as non-coding (Figure 6L) sequences. We refer to this ectopic DNA elimination phenomenon as co-deletion (coDel). Because coDel often removed regions wider than the target, Late-scnRNAs most likely further induce Late-scnRNA production at the target loci in *cis*, as predicted in Figure 6E ("v"). Because the assay relies on transformation of *Tetrahymena* cells, which occurs inefficiently (roughly 1 out of 10,000 cells), we lack the sensitivity to detect Late-scnRNAs from a target sequence during a coDel process. However, the fact that coDel was suppressed in *TWI11* MIC-KO cells (Figure 6M) argues that coDel is mediated by Late-scnRNAs.

The coDel phenomenon not only supports the *cis* triggering of Late-scnRNA production by Early-scnRNAs but also indicates that (1) Late-scnRNAs can further target their complementary sequences in *trans* (2° recognition) for DNA elimination (because some Type-B IESs do not express Late-scnRNAs in *TWI1*/*TWI11* MIC-KO [Figure 2J, arrows], expression of Late-scnRNAs from these Type-B IESs may be triggered only by 2° recognition with Late-scnRNAs produced from other Type-B IESs); (2) the prerequisite for becoming the target of 2° recognition is either highly relaxed or nonexistent because three randomly chosen target sequences induced ectopic DNA elimination (Figures 6F, 6K, and 6L); and (3) unlike Early-scnRNAs (Aronica et al., 2008; Schoeberl et al., 2012), Late-scnRNAs complementary to the parental MAC genome escape degradation, most likely because Twi1p/Twi11p-Late-scnRNA complexes do not localize to the parental MAC (see Figure S3 for Twi11p and Noto et al., 2010 for Twi1p).

Heterochromatin Is Required for Late-scnRNA Production

We assumed that the *cis* triggering of Late-scnRNA production by Early-scnRNAs might be mediated by the production of double-stranded RNAs through RNA-dependent RNA polymerase (RdRP) or by heterochromatin, which spreads along chromatin in many eukaryotes.

Rdr1p, the single RdRP in *Tetrahymena*, is essential for vegetative growth (Lee et al., 2009), and thus, the *RDR1* MAC&MIC-KO is lethal. We instead analyzed the accumulation of scnRNAs in *RDR1* MIC-KO cells and found that Late-scnRNAs were accumulated normally in the absence of zygotic expression of Rdr1p (Figure 2M). Therefore, although we cannot exclude the possibility that maternally expressed Rdr1p is sufficient to produce normal amounts of Late-scnRNAs, it is likely that Rdr1p is dispensable for the Late-scnRNA biogenesis.

The interaction of Early-scnRNA with new MAC chromatin induces heterochromatin formation, including the accumulation of H3K9/K27me and its interacting HP1-like protein Pdd1p (Liu

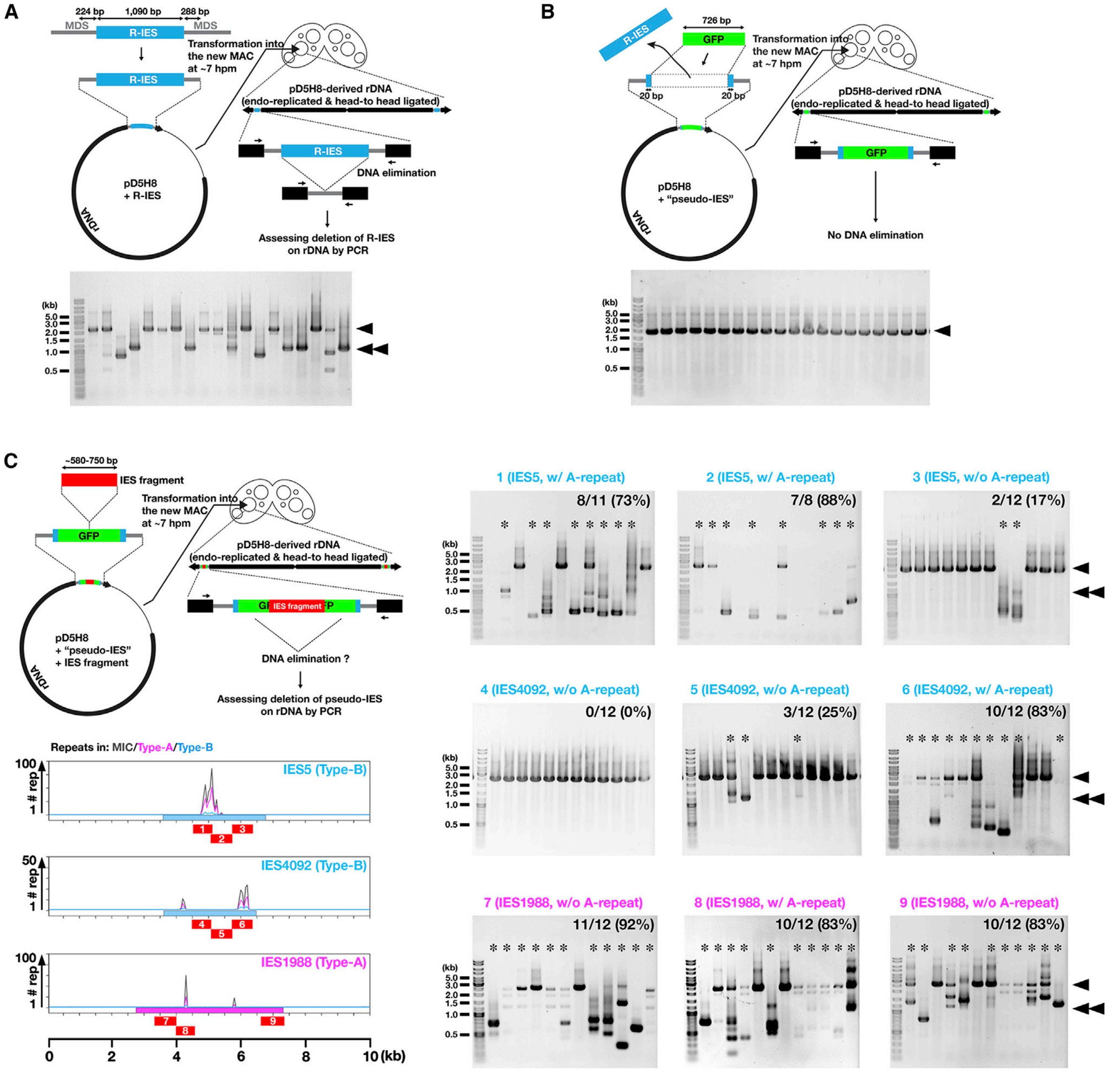

Figure 5. Role of A-Repeats in DNA Elimination

(A) DNA elimination assay of R-IES. R-IES (a Type-B IES) and its flanking MDS regions was inserted into the extra-chromosomal vector pD5H8 and introduced into the developing MAC. Progeny lines were established and elimination of R-IES on the vector was analyzed by PCR using primers complementary to the vector (arrows). The PCR results are shown on the bottom. The arrowhead and double arrowhead indicate the expected position of the PCR products from R-IES loci on the vector without or with R-IES elimination, respectively.

(B) DNA elimination assay of a pseudo-IES. Most of the R-IES in pD5H8 shown in (A) was replaced by a GFP-coding sequence and analyzed as in (A).

(C) DNA elimination assays assessing the DNA elimination-inducing activities of IES fragments. A fragment of an IES of interest (red boxes with numbers) was inserted into the middle of the pseudo-IES shown in (B) and tested to see if the fragment restored DNA elimination of the pseudo-IES. The progeny cell lines showing any deletion at the pseudo-IES loci (marked with asterisks) were counted. The arrowheads indicate PCR products from the pseudo-IES loci on the vector without any DNA elimination. The double arrowheads show the expected positions of PCR products from the pseudo-IES loci on the vector following DNA elimination exactly at the borders of the original R-IES.

et al., 2007; Taverna et al., 2002). We found that *EZL1*, which encodes H3K9/K27 methyltransferase, and *PDD1* were necessary for the production of Late-scnRNAs (Figures 2O and 2Q), although they were not required for the accumulation of Early-scnRNAs (Figures 2N and 2P). Therefore, we conclude that heterochromatin components are required for Late-scnRNA

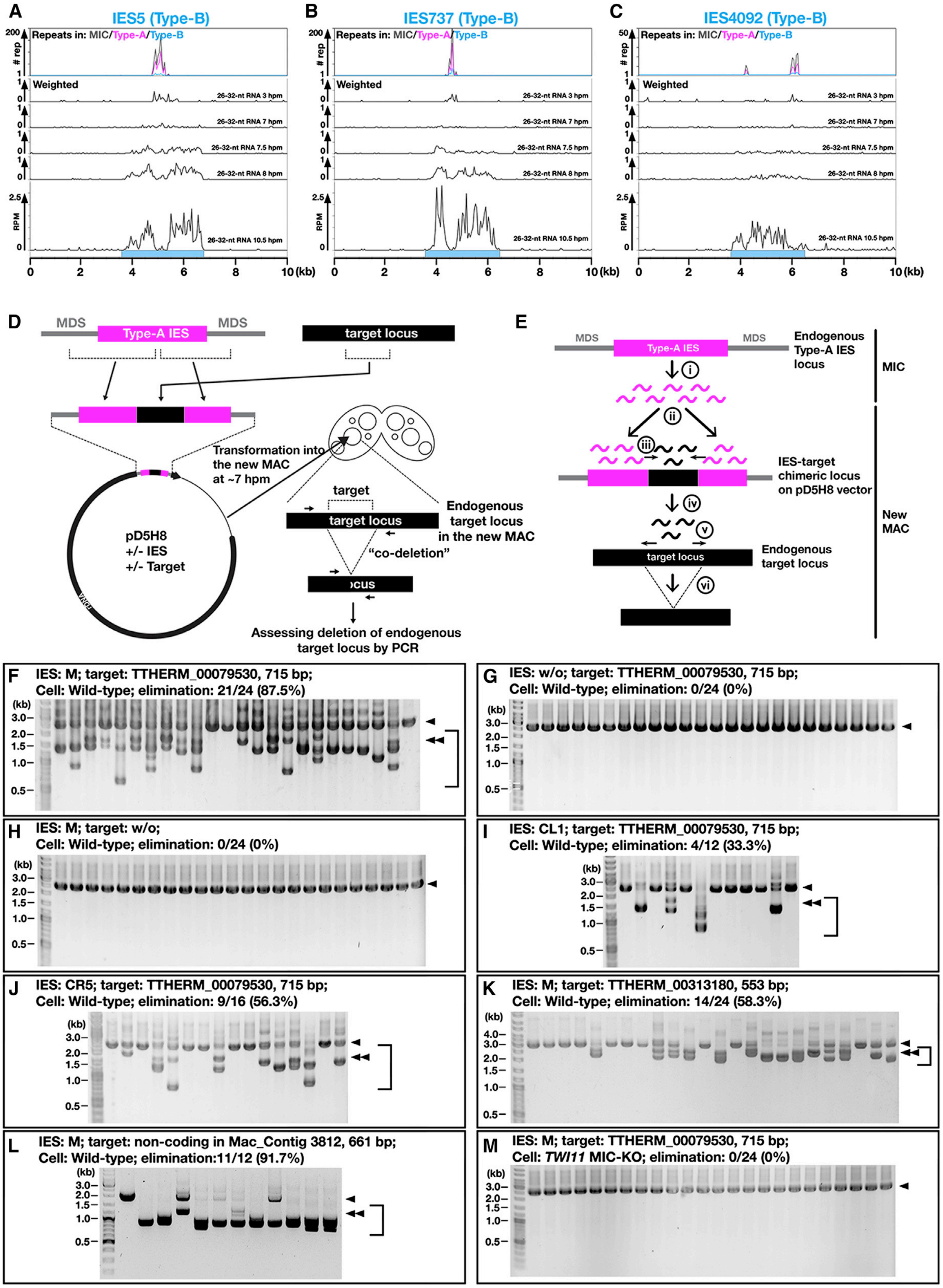

(legend on next page)

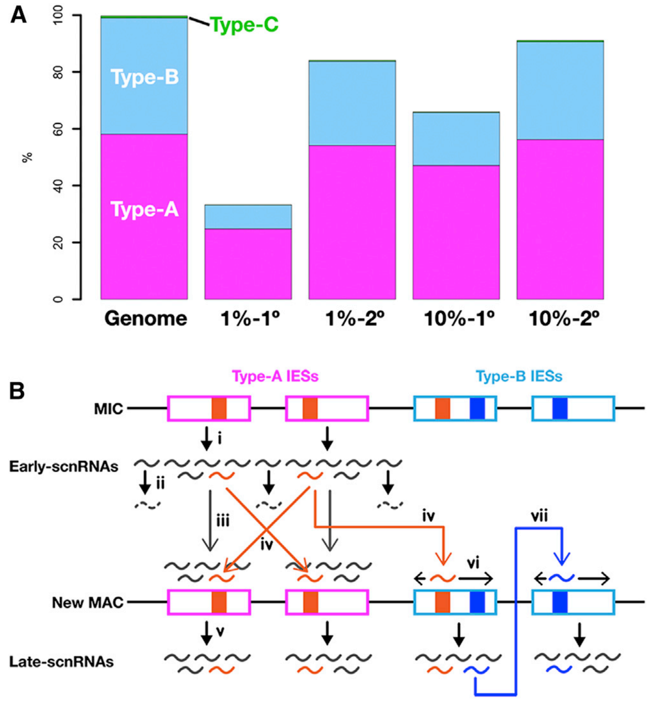

Figure 7. A *trans*-Recognition Network in IES Recognition

(A) Results of a simulation of accidental loss of Early-scnRNA expression. See the text for details. The average fractions of 1° and 2° IESs in each condition, with three different sets of randomly chosen seed Type-A IESs (47 and 469 Type-A IESs for 1% and 10% seeds, respectively) are shown.

(B) A model for IES recognition. Early-scnRNAs are expressed from Type-A IESs (magenta boxes) and their flanking sequences in the MIC (i), and the latter are degraded in the parental MAC (ii). In the new MAC, Early-scnRNAs recognize the Type-A IESs from which they are derived (iii) as well as other Type-A and Type-B (sky-blue boxes) IESs in *trans* (iv) through A-repeats (filled orange boxes) to trigger Late-scnRNA production (v). In an IES, regions producing Late-scnRNAs spread in *cis* (vi). Late-scnRNAs further *trans*-recognize other IESs (vii).

production, and *cis* triggering of Late-scnRNA production is most likely mediated by the *cis* spreading of heterochromatin on IESs.

trans Recognition and *cis* Spreading Provide Robustness in DNA Elimination

We tested the robustness of the DNA elimination system by simulating the accidental loss of Early-scnRNA expression from a majority of Type-A IESs. We randomly chose 47 (1%) or 469 (10%) non-redundant Type-A IESs, and all possible 20-mers were extracted from the chosen Type-A IESs. Then, IESs in which there are more than 250 complementary bases to the 20-mers in one of the possible 500-nt windows were identified as recognized IESs (1° IESs). Next, all possible 20-mers were extracted from the 1° IESs, and the same calculation was repeated to identify 2° IESs. The determination of the threshold for IES recognition in the simulation is based on two previous observations: (1) an ~300-nt segment of the M-IES (a Type-A IES) is sufficient to induce its DNA elimination (Kowalczyk et al., 2006), and (2) for many small RNA-target interactions, the seed sequence, which typically spans positions 2–8 from the 5′ end, plays a more important role than the rest of the small RNA sequences. Therefore, we believe the target recognition criteria above gives a conservative prediction of target genomic loci.

The simulation of accidental loss of Early-scnRNA expression (Figure 7A) suggests that even if Early-scnRNAs are expressed only from 1% of Type-A IESs, they could recognize 33% of all IESs (1% to 1°), and if Late-scnRNAs derived from these 1° IESs also function in *trans* recognition, they could identify up to 84% of total IESs (1% to 2°). If 10% of Type-A IESs express Early-scnRNAs, such 1° and 2° IESs could increase coverage to 66% (10% to 1°) and 91% (10% to 2°), respectively. These results indicate that Early-scnRNAs from a small set of Type-A IESs are sufficient to identify most of the other IESs, demonstrating strong robustness of the DNA elimination mechanism.

Next, to test the specificity of the *trans*-recognition system, we queried whether genic sequences are targeted by IES-derived scnRNAs in *trans*. By applying the target recognition criteria used above, we determined how many gene-coding sequences can be targeted if all of the IESs express scnRNAs homogeneously and if all of the expressed scnRNAs are involved in *trans* recognition. Out of 20,491 predicted gene-coding sequences used for the simulation, we found that only 312 (1.3%) were predicted to be *trans*-targeted by IES-derived scnRNAs. Therefore, gene-coding sequences are generally devoid of a *trans*-recognition system.

DISCUSSION

In this study, we demonstrated that there is an unanticipated mechanism regulating programmed DNA elimination: a genome-wide *trans*-recognition network for IES identification. In this mechanism (Figure 7B), Early-scnRNAs produced from Type-A IESs in the MIC (i) identify not only the IESs from which they are derived (iii) but also other IESs in *trans* (iv) to trigger the *cis* spreading of Late-scnRNA production in the IESs (vi). This *cis* spreading of Late-scnRNA production requires heterochromatin formation (Figures 2O and 2Q). Furthermore, these Late-scnRNAs can recognize other IESs in *trans* (vii). This "chain

Figure 6. Co-deletion

(A–C) Normalized and weighted numbers of sequenced total 26- to 32-nt small RNAs from wild-type cells at indicated time points mapping to the representative Type-B IES loci are shown as histograms with 50-nt bins. Sky-blue boxes indicate IESs.

(D and E) Schematic drawings of the co-deletion (coDel) experiment (D) and of the hypothetical actions of scnRNAs in coDel (E). See the text for details.

(F–M) Indicated IESs and target loci were cloned into the extra-chromosomal vector pD5H8 and introduced into the new MACs of conjugating wild-type (F–L) or *TWI11* MIC-KO (M) cells. Deletions at the endogenous target loci in progeny cells were analyzed by PCR as in (D). For the "w/o target" experiment (H), the TTHERM_00079350 locus was analyzed. The number of progeny lines showing any deletions at the endogenous target loci was determined. The arrowheads and brackets indicate PCR products from the target loci without or with deletions, respectively. The double arrowheads show the expected positions of PCR products from the target loci with deletions exactly corresponding to the target sequences.

See also Figure S3.

reaction" of Late-scnRNA production by the *trans*-recognition network most likely provides strong robustness in DNA elimination by buffering cell-to-cell variability in the initial Early-scnRNA populations (Figure 7A).

Conceptually, the *trans*-recognition network for IES identification in *Tetrahymena* and the piRNA pathway in flies and mammals are strategically similar: Type-A IESs may be the functional equivalents of piRNA clusters, as both contain remnants of TEs and produce small RNAs for the *trans* recognition of TE-related sequences across the genome (Aravin et al., 2007; Brennecke et al., 2007). Furthermore, similar to "ping-pong" amplification of piRNAs by the feed-forward endonucleolytic cycle (Brennecke et al., 2007; Gunawardane et al., 2007), Early-scnRNAs trigger a chain reaction of Late-scnRNA production by *trans* recognition and *cis* spreading, and this reaction is likely mediated by heterochromatin. Because the pattern of DNA elimination in the parent is the basis of DNA elimination in ciliate progeny, robust DNA elimination by the *trans*-recognition network would reinforce faithful transgenerational transmission of TE memory, just as piRNA amplification fuels the maternal transmission of piRNAs to silence TEs (Brennecke et al., 2008). Because DNA elimination in *Tetrahymena* occurs within a Dicer-dependent siRNA pathway, the two conceptually similar *trans*-recognition network-type TE surveillance systems, the piRNA pathway in metazoans and DNA elimination in *Tetrahymena,* are products of evolutionary convergence. It is very interesting that such TE surveillance can be achieved by two very distinct small RNA mechanisms. Because TEs are found in almost all eukaryotes, such systems might have evolved multiple times in different eukaryotic linages.

A similar small RNA amplification cycle has been proposed for iesRNAs in *Paramecium* (Sandoval et al., 2014). However, *Paramecium* IESs are mostly unique sequences (Arnaiz et al., 2012), and therefore, iesRNAs are most likely not involved in *trans*-recognition between different IESs. Instead, iesRNAs from one genome in the polycopy developing MAC probably help to recognize another copy of the same IESs to ensure complete DNA elimination (Sandoval et al., 2014). Recognition of the same IESs by Late-scnRNAs might serve a similar function in *Tetrahymena,* as DNA elimination of some Type-A IESs was mildly affected in *TWI1*/*TWI11* MIC-KO cells (Figure 3H), in which Late-scnRNAs are unstable (Figures 2J and 2K).

Misregulation of the *trans*-recognition network accompanied by a chain reaction of Late-scnRNA production could potentially cause elimination of important genomic regions adjacent to IESs and lead to runaway Late-scnRNA amplification, resulting in the elimination of regions distal to IESs. Therefore, the *cis* triggering of Late-scnRNA production must occur with high precision within the IES borders. The previously characterized "*cis*-acting sequences" (Chalker et al., 1999; Godiska et al., 1993) might supply this boundary information.

Regardless of the nature of boundary information, it is difficult to imagine that newly invaded transposons are always associated with such regulatory sequences. During the coDel processes, DNA elimination boundaries occur mostly within ~0.5–1 kb from target sequences (Figure 6), indicating that a system is likely in place to limit *cis* spreading, even at non-IES loci. Certain DNA sequences that insulate *cis* spreading may frequently occur throughout the genome, and *cis* spreading might always be limited within two such "insulator" sequences. Alternatively, because Type-A IESs, which we believe are "young" IESs, have markedly higher GC content than the other IESs as well as the entire MIC genome (Figure 3D), the difference in base compositions between TEs and the host genome may provide an insulator function. Nonetheless, because coDel occurs with heterogeneous boundaries (Figure 6), only authentic IESs or their flanking sequences should contain information that tightly restricts the *cis* triggering of Late-scnRNA production within IESs and thus maintains precise DNA elimination boundaries. It is possible that only the sequences associated with such boundary information have been selected through evolution to be IESs. Future studies should be designed to identify the molecular nature of the essential boundary information that allows the *trans*-recognition network to strictly distinguish TE-related sequences from the rest of the genome.

EXPERIMENTAL PROCEDURES

Materials and Bioinformatic Analyses

Tetrahymena strains, antibodies, oligonucleotides, and bioinformatic analyses (distributions of IESs, CDSs, TEs and repeats, IES classification, and simulation of accidental loss of Early-scnRNA expression and targeting of protein coding sequences) are described in Supplemental Experimental Procedures. The draft MIC genome sequence (version 2) was obtained from the Tetrahymena Comparative Sequencing Project (Broad Institute of Harvard and MIT).

Small RNA Analyses

Small RNA purification, co-immunoprecipitation, high-throughput sequencing, and analyses by gel electrophoresis were performed as previously described (Noto et al., 2010; Schoeberl et al., 2012).

Purification of the New MAC and DNA Elimination Analysis

New MACs from exconjugants at 36 hpm were purified by fluorescence-activated cell sorting, and genomic DNA libraries were generated and sequenced using a HiSeq2000 platform with 50-nt single reads. To obtain RIs, the number of normalized sequence reads from purified new MACs mapping to each IES was calculated and divided by the number of reads that map the flanking MAC-destined sequences of the IES (1-kb sequences located 500 bp upstream and downstream of the IES). Detailed procedures are described in Supplemental Experimental Procedures.

DNA Elimination Analysis with Pseudo-IES

Fragments of IESs were cloned into the NotI site of pD5H8-pseudo-IES. The pD5H8-R-IES, pD5H8-pseudo-IES and pD5H8-pseudo-IES containing the IES fragments were introduced into conjugating cells (7 hpm) and cultured in 10 mM Tris (pH 7.5) overnight, and cells were selected with paromomycin. DNA elimination in the rDNA vector was analyzed by genomic DNA PCR. Detailed procedures are described in Supplemental Experimental Procedures.

coDel

The vectors pMcoDel, pCL1coDel, and pCR5coDel were digested with NotI, and target sequences were inserted. The vectors were introduced into WT or *TWI11* MIC-KO cells as described above, and DNA eliminations at the endogenous target loci were analyzed by genomic DNA PCR. Detailed procedures are described in Supplemental Experimental Procedures.

ACCESSION NUMBERS

The accession numbers for the datasets reported in this paper are GEO: GSE68455, GSE68457.

SUPPLEMENTAL INFORMATION

Supplemental Information includes Supplemental Experimental Procedures, three figures, and two tables and can be found with this article online at http://dx.doi.org/10.1016/j.molcel.2015.05.024.

AUTHOR CONTRIBUTIONS

T.N., K.K., J.H.S., M.A.G., and K.M. designed the experiments; T.N., K.K., J.H.S., A.H., K.J.W., and K.M. performed the experiments; and T.N., K.K., and K.M. prepared the manuscript.

ACKNOWLEDGMENTS

We wish to thank Eduardo Orias for his comments. This work was supported by the Austrian Academy of Sciences, the European Community's 7th Framework Program (ERC Starting Grant 204986), and the Austrian Science Fund (P26032-B22; F4307-B09). K.K. was supported by a Naito Memorial Grant for Research Abroad from the Naito Foundation. K.J.W. was supported by an EMBO Long-Term Fellowship.

Received: November 24, 2014
Revised: March 6, 2015
Accepted: May 15, 2015
Published: June 18, 2015

REFERENCES

Aravin, A.A., Sachidanandam, R., Girard, A., Fejes-Toth, K., and Hannon, G.J. (2007). Developmentally regulated piRNA clusters implicate MILI in transposon control. Science *316*, 744–747.

Arnaiz, O., Mathy, N., Baudry, C., Malinsky, S., Aury, J.M., Denby Wilkes, C., Garnier, O., Labadie, K., Lauderdale, B.E., Le Mouël, A., et al. (2012). The *Paramecium* germline genome provides a niche for intragenic parasitic DNA: evolutionary dynamics of internal eliminated sequences. PLoS Genet. *8*, e1002984.

Aronica, L., Bednenko, J., Noto, T., DeSouza, L.V., Siu, K.W., Loidl, J., Pearlman, R.E., Gorovsky, M.A., and Mochizuki, K. (2008). Study of an RNA helicase implicates small RNA-noncoding RNA interactions in programmed DNA elimination in *Tetrahymena*. Genes Dev. *22*, 2228–2241.

Austerberry, C.F., Allis, C.D., and Yao, M.C. (1984). Specific DNA rearrangements in synchronously developing nuclei of *Tetrahymena*. Proc. Natl. Acad. Sci. USA *81*, 7383–7387.

Brennecke, J., Aravin, A.A., Stark, A., Dus, M., Kellis, M., Sachidanandam, R., and Hannon, G.J. (2007). Discrete small RNA-generating loci as master regulators of transposon activity in *Drosophila*. Cell *128*, 1089–1103.

Brennecke, J., Malone, C.D., Aravin, A.A., Sachidanandam, R., Stark, A., and Hannon, G.J. (2008). An epigenetic role for maternally inherited piRNAs in transposon silencing. Science *322*, 1387–1392.

Chalker, D.L., La Terza, A., Wilson, A., Kroenke, C.D., and Yao, M.C. (1999). Flanking regulatory sequences of the *Tetrahymena* R deletion element determine the boundaries of DNA rearrangement. Mol. Cell. Biol. *19*, 5631–5641.

Chalker, D.L., Meyer, E., and Mochizuki, K. (2013). Epigenetics of ciliates. Cold Spring Harb. Perspect. Biol. *5*, a017764.

Chen, X., Bracht, J.R., Goldman, A.D., Dolzhenko, E., Clay, D.M., Swart, E.C., Perlman, D.H., Doak, T.G., Stuart, A., Amemiya, C.T., et al. (2014). The architecture of a scrambled genome reveals massive levels of genomic rearrangement during development. Cell *158*, 1187–1198.

Couvillion, M.T., Lee, S.R., Hogstad, B., Malone, C.D., Tonkin, L.A., Sachidanandam, R., Hannon, G.J., and Collins, K. (2009). Sequence, biogenesis, and function of diverse small RNA classes bound to the Piwi family proteins of *Tetrahymena thermophila*. Genes Dev. *23*, 2016–2032.

Coyne, R.S., Stover, N.A., and Miao, W. (2012). Whole genome studies of *Tetrahymena*. Methods Cell Biol. *109*, 53–81.

Dumesic, P.A., Natarajan, P., Chen, C., Drinnenberg, I.A., Schiller, B.J., Thompson, J., Moresco, J.J., Yates, J.R., 3rd, Bartel, D.P., and Madhani, H.D. (2013). Stalled spliceosomes are a signal for RNAi-mediated genome defense. Cell *152*, 957–968.

Eisen, J.A., Coyne, R.S., Wu, M., Wu, D., Thiagarajan, M., Wortman, J.R., Badger, J.H., Ren, Q., Amedeo, P., Jones, K.M., et al. (2006). Macronuclear genome sequence of the ciliate *Tetrahymena thermophila*, a model eukaryote. PLoS Biol. *4*, e286.

Fang, W., Wang, X., Bracht, J.R., Nowacki, M., and Landweber, L.F. (2012). Piwi-interacting RNAs protect DNA against loss during *Oxytricha* genome rearrangement. Cell *151*, 1243–1255.

Fillingham, J.S., Thing, T.A., Vythilingum, N., Keuroghlian, A., Bruno, D., Golding, G.B., and Pearlman, R.E. (2004). A non-long terminal repeat retrotransposon family is restricted to the germ line micronucleus of the ciliated protozoan *Tetrahymena thermophila*. Eukaryot. Cell *3*, 157–169.

Godiska, R., and Yao, M.C. (1990). A programmed site-specific DNA rearrangement in *Tetrahymena thermophila* requires flanking polypurine tracts. Cell *61*, 1237–1246.

Godiska, R., James, C., and Yao, M.C. (1993). A distant 10-bp sequence specifies the boundaries of a programmed DNA deletion in *Tetrahymena*. Genes Dev. *7* (12A), 2357–2365.

Gunawardane, L.S., Saito, K., Nishida, K.M., Miyoshi, K., Kawamura, Y., Nagami, T., Siomi, H., and Siomi, M.C. (2007). A slicer-mediated mechanism for repeat-associated siRNA 5′ end formation in *Drosophila*. Science *315*, 1587–1590.

Guzzardo, P.M., Muerdter, F., and Hannon, G.J. (2013). The piRNA pathway in flies: highlights and future directions. Curr. Opin. Genet. Dev. *23*, 44–52.

Kowalczyk, C.A., Anderson, A.M., Arce-Larreta, M., and Chalker, D.L. (2006). The germ line limited M element of *Tetrahymena* is targeted for elimination from the somatic genome by a homology-dependent mechanism. Nucleic Acids Res. *34*, 5778–5789.

Kurth, H.M., and Mochizuki, K. (2009). 2′-O-methylation stabilizes Piwi-associated small RNAs and ensures DNA elimination in *Tetrahymena*. RNA *15*, 675–685.

Lee, S.R., Talsky, K.B., and Collins, K. (2009). A single RNA-dependent RNA polymerase assembles with mutually exclusive nucleotidyl transferase subunits to direct different pathways of small RNA biogenesis. RNA *15*, 1363–1374.

Lee, N.N., Chalamcharla, V.R., Reyes-Turcu, F., Mehta, S., Zofall, M., Balachandran, V., Dhakshnamoorthy, J., Taneja, N., Yamanaka, S., Zhou, M., and Grewal, S.I. (2013). Mtr4-like protein coordinates nuclear RNA processing for heterochromatin assembly and for telomere maintenance. Cell *155*, 1061–1074.

Levin, H.L., and Moran, J.V. (2011). Dynamic interactions between transposable elements and their hosts. Nat. Rev. Genet. *12*, 615–627.

Liu, Y., Taverna, S.D., Muratore, T.L., Shabanowitz, J., Hunt, D.F., and Allis, C.D. (2007). RNAi-dependent H3K27 methylation is required for heterochromatin formation and DNA elimination in *Tetrahymena*. Genes Dev. *21*, 1530–1545.

Malone, C.D., Anderson, A.M., Motl, J.A., Rexer, C.H., and Chalker, D.L. (2005). Germ line transcripts are processed by a Dicer-like protein that is essential for developmentally programmed genome rearrangements of *Tetrahymena thermophila*. Mol. Cell. Biol. *25*, 9151–9164.

Mochizuki, K., and Gorovsky, M.A. (2004). RNA polymerase II localizes in *Tetrahymena thermophila* meiotic micronuclei when micronuclear transcription associated with genome rearrangement occurs. Eukaryot. Cell *3*, 1233–1240.

Mochizuki, K., and Gorovsky, M.A. (2005). A Dicer-like protein in *Tetrahymena* has distinct functions in genome rearrangement, chromosome segregation, and meiotic prophase. Genes Dev. *19*, 77–89.

Mochizuki, K., and Kurth, H.M. (2013). Loading and pre-loading processes generate a distinct siRNA population in *Tetrahymena*. Biochem. Biophys. Res. Commun. *436*, 497–502.

Mochizuki, K., Fine, N.A., Fujisawa, T., and Gorovsky, M.A. (2002). Analysis of a *piwi*-related gene implicates small RNAs in genome rearrangement in *tetrahymena*. Cell *110*, 689–699.

Noto, T., Kurth, H.M., Kataoka, K., Aronica, L., DeSouza, L.V., Siu, K.W., Pearlman, R.E., Gorovsky, M.A., and Mochizuki, K. (2010). The *Tetrahymena* argonaute-binding protein Giw1p directs a mature argonaute-siRNA complex to the nucleus. Cell *140*, 692–703.

Sandoval, P.Y., Swart, E.C., Arambasic, M., and Nowacki, M. (2014). Functional diversification of Dicer-like proteins and small RNAs required for genome sculpting. Dev. Cell *28*, 174–188.

Saveliev, S.V., and Cox, M.M. (1994). The fate of deleted DNA produced during programmed genomic deletion events in *Tetrahymena thermophila*. Nucleic Acids Res. *22*, 5695–5701.

Schoeberl, U.E., Kurth, H.M., Noto, T., and Mochizuki, K. (2012). Biased transcription and selective degradation of small RNAs shape the pattern of DNA elimination in *Tetrahymena*. Genes Dev. *26*, 1729–1742.

Seth, M., Shirayama, M., Gu, W., Ishidate, T., Conte, D., Jr., and Mello, C.C. (2013). The *C. elegans* CSR-1 argonaute pathway counteracts epigenetic silencing to promote germline gene expression. Dev. Cell *27*, 656–663.

Siomi, M.C., Sato, K., Pezic, D., and Aravin, A.A. (2011). PIWI-interacting small RNAs: the vanguard of genome defence. Nat. Rev. Mol. Cell Biol. *12*, 246–258.

Slotkin, R.K., Vaughn, M., Borges, F., Tanurdzić, M., Becker, J.D., Feijó, J.A., and Martienssen, R.A. (2009). Epigenetic reprogramming and small RNA silencing of transposable elements in pollen. Cell *136*, 461–472.

Taverna, S.D., Coyne, R.S., and Allis, C.D. (2002). Methylation of histone h3 at lysine 9 targets programmed DNA elimination in *tetrahymena*. Cell *110*, 701–711.

Wedeles, C.J., Wu, M.Z., and Claycomb, J.M. (2013). Protection of germline gene expression by the *C. elegans* Argonaute CSR-1. Dev. Cell *27*, 664–671.

Woehrer, S.L., Aronica, L., Suhren, J.H., Busch, C.J., Noto, T., and Mochizuki, K. (2015). A *Tetrahymena* Hsp90 co-chaperone promotes siRNA loading by ATP-dependent and ATP-independent mechanisms. EMBO J. *34*, 559–577.

Wuitschick, J.D., Gershan, J.A., Lochowicz, A.J., Li, S., and Karrer, K.M. (2002). A novel family of mobile genetic elements is limited to the germline genome in *Tetrahymena thermophila*. Nucleic Acids Res. *30*, 2524–2537.

Yao, M.C., Fuller, P., and Xi, X. (2003). Programmed DNA deletion as an RNA-guided system of genome defense. Science *300*, 1581–1584.

Molecular Cell

Supplemental Information

Small-RNA-Mediated Genome-wide *trans*-Recognition Network in *Tetrahymena* DNA Elimination

Tomoko Noto, Kensuke Kataoka, Jan H. Suhren, Azusa Hayashi, Katrina J. Woolcock, Martin A. Gorovsky, and Kazufumi Mochizuki

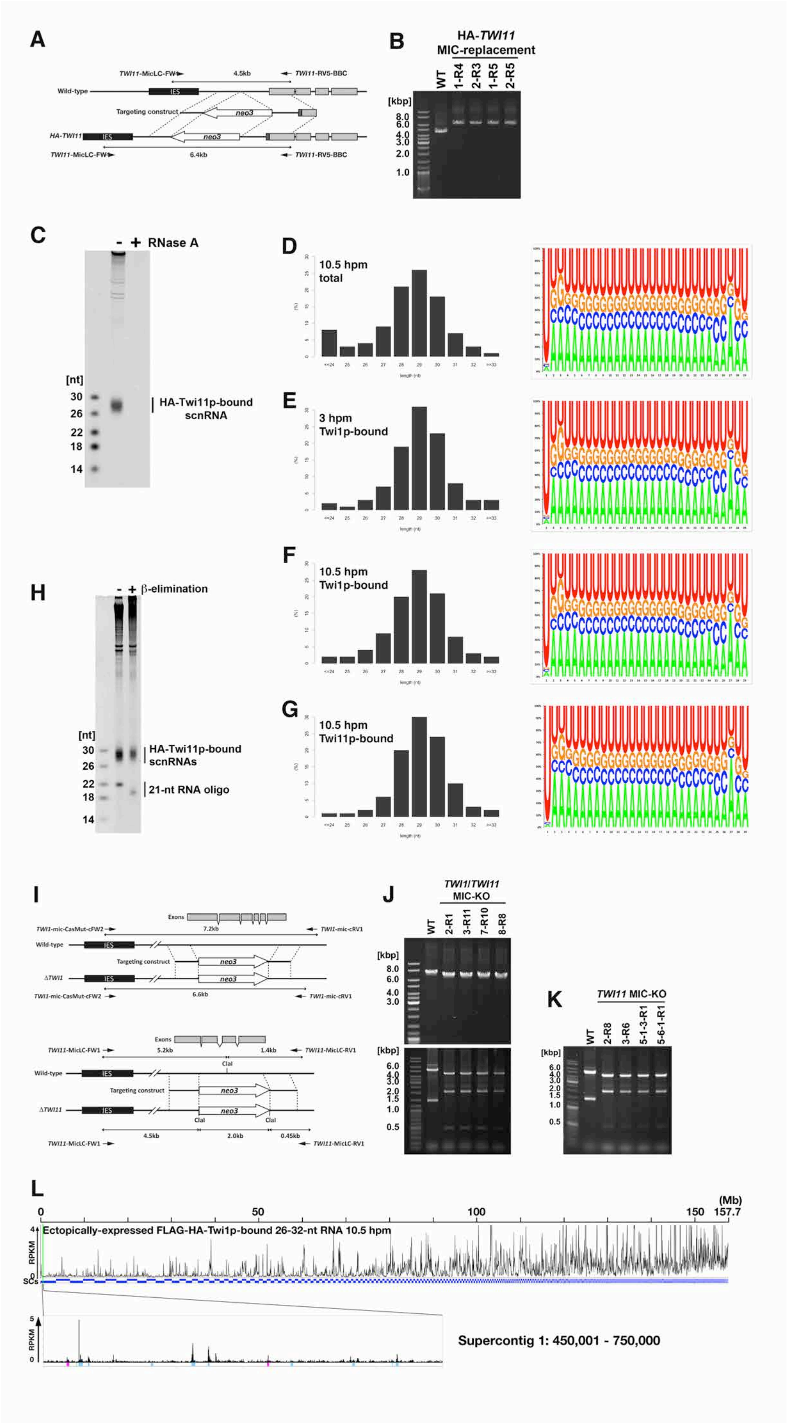

Supplementary Figure S1 (related to Figure 2). Analyses of Twi1p and Twi11p-bound small RNAs.

(A) Diagrams of the wild-type TWI11 and HA-TWI11 MIC loci.
(B) Genomic PCR demonstrating the replacement of endogenous MIC TWI11 genes with the HA-TWI11 construct. Total DNA isolated from the indicated strains was used for PCR with the primers shown in (A). One of the primers was complementary to IESs, and thus, PCR amplified only the loci in the MIC.
(C) HA-Twi11p-containing complexes were immunopurified with anti-HA antibody from the strains in which the endogenous TWI11 loci in the MIC were replaced with HA-TWI11 gene (HA-TWI11) at 10.5 hpm. Co-purified RNAs were incubated with (+) or without (-) RNase A, separated on a denaturing gel and stained with a nucleic-acid specific dye. Positions of single-stranded RNA markers are indicated on the left.
(D-G) Size distributions of total small RNAs (left) and base composition of 29-nt scnRNAs (right) from wild-type cells at 10.5 hpm (D), Twi1p-bound RNAs from wild-type cells at 3 hpm (Early-scnRNAs, E), Twi1p-bound RNAs from wild-type cells at 10.5 hpm (F), and Twi11p-bound RNAs from wild-type cells at 10.5 hpm (Late-scnRNAs, G).
(H) HA-Twi11p-bound RNAs were prepared as in (C), mixed with a synthetic, 21-nt RNA and subjected to periodate oxidation/β-elimination (+). The reactions were separated in a denaturing gel and visualized using a nucleic acid-specific dye. Untreated RNAs (-) were analyzed in the same gel. The positions of the RNA markers are shown on the left. The mobility of the HA-Twi11p-bound small RNAs did not increase after the reaction, whereas the mobility of the unmodified synthetic 21-nt RNA did, indicating that Twi11p-bound Late-scnRNAs were modified at their 3' terminal nucleotides.
(I) Diagrams of the wild-type TWI1 and TWI1 KO (ΔTWI1) loci (top) and the wild-type TWI11 and TWI11 KO (ΔTWI11) MIC loci (bottom).
(J, K) Genomic PCR demonstrating the replacement of endogenous TWI1 and TWI11 genes by the KO constructs in TWI1/TWI11 double MIC-KO (J) and TWI11 (single) MIC-KO cells (K). Total DNA isolated from the indicated strains was used for PCR with the primers shown in (I). For both loci, one of the primers was complementary to IESs, and thus, PCR amplified only the loci in the MIC. PCR products from the TWI11 locus were digested with ClaI.
(L) Normalized numbers (RPKM) of uniquely mapped 26-32-nt RNAs immunopurified with FLAG-HA-Twi1p, which was ectopically expressed from 6 hpm and immunoprecipitated at 10.5 hpm, are shown as histograms with 50-kb (left) or 100-b (right) bins. The 300-kb window on the right is marked with a green line. Colored boxes below the right histograms indicate IES positions (magenta: Type-A, sky-blue: Type-B; see Figure 3 for the IES classification).

Noto et al. Supplementary Figure S1

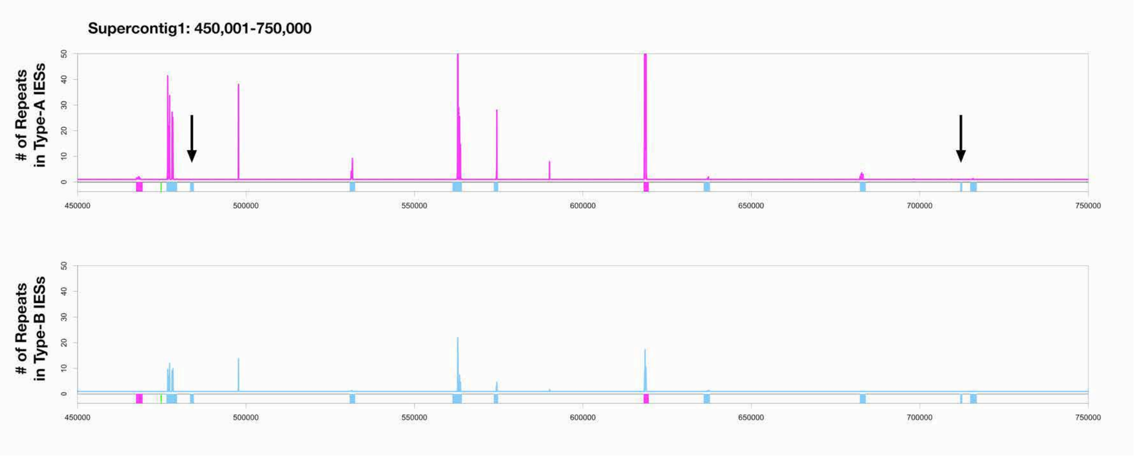

Supplementary Figure S2 (related to Figure 4). Distributions of sequences complementary to IESs.

Distributions of sequences complementary to IESs in a B-region of the MIC genome (300 kb). All possible 25-nt sequences were extracted from all Type-A IESs (magenta, top) and Type-B IESs (sky-blue, bottom). Their occurrence in given genomic locations was calculated and shown as histograms with 100-nt bins. Positions of Type-A and Type-B IESs are marked with magenta and sky-blue boxes, respectively. Type-B IESs possessing no repeat sequences are marked with arrows.

Noto et al. Supplementary Figure S2

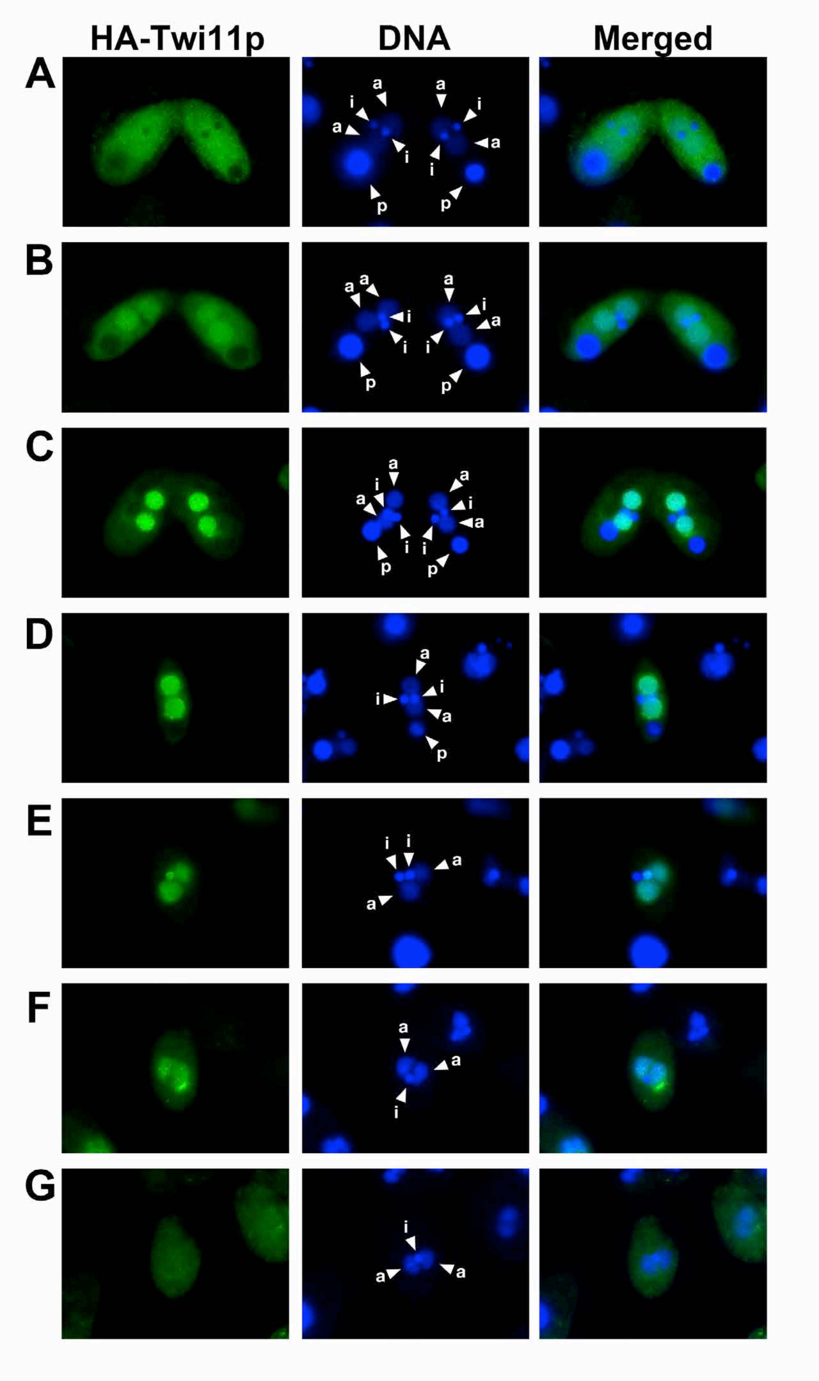

Supplementary Figure S3 (related to Figure 6). Localization of HA-Twi11p.

The localization of HA-Twi11p during late conjugation and exconjugation stages in strains in which the endogenous TWI11 loci in the MIC were replaced with the HA-TWI11 gene (HA-TWI11) was analyzed by indirect immunofluorescent staining using an anti-HA antibody (green). DNA was counterstained with DAPI (blue). Cells were in the Nuclear Alignment stage (A-C) or Exconjugation stage (D-G). HA-Twi11p first appears in the cytoplasm at the Nuclear Alignment stage and then moves into the new MAC, where it remains until the Exconjugation stages. HA-Twi11p also localizes to the MIC during the Exconjugation stage (E), although the significance of this MIC localization is not clear. The parental (old) MACs, the new MACs, and the new MICs are marked by arrowheads labeled “p," “a” and “i," respectively.

Supplemental Experimental Procedures

Tetrahymena Strains

Wild-type B2086 and CU428 strains of *Tetrahymena thermophila* were obtained from the *Tetrahymena* Stock Center at Cornell University. The *TWI1* MAC-knockout (KO), *FLAG-HA-TWI1, DCL1* MIC KO, and *EZL1* MAC&MIC KO strains were previously described (Aronica et al., 2008; Mochizuki et al., 2002; Mochizuki and Gorovsky, 2005; Noto et al., 2010). The construction of the *HA-TWI11*, *TWI11* MIC-KO, *TWI1/TWI11* double MIC-KO, *MTT1*::FLAG-HA-*TWI1,* and *RDR1* MIC KO strains is described below. Cells were grown in SPP medium (Gorovsky et al., 1975) containing 2% proteose peptone at 30 °C. For conjugation, growing cells (~5-7 x 10^5/ml) of two different mating types were washed, pre-starved (~12-24 hr) and mixed in 10 mM Tris (pH 7.5) at 30 °C.

Constructions of gene knockout and transgenic strains

To make the *HA-TWI11* construct (Supplementary Figure S1A), an HA coding sequence was inserted immediately after the start codon of *TWI11* by overlapping PCR. The HA sequence, followed by the N-terminal segment of *TWI11*, was amplified with the *TWI11*-FW9-HA and *TWI11*-RV12-Xho primers (Product-A). Part of the 5' flanking sequences, the 5'-UTR, the start codon, and the HA sequences were amplified by the first PCR using *TWI11*-FW7-Spe + *TWI11*-VR7-EBS (Product-B) and *TWI11*-FW8-EBS + *TWI11*-RV8-HA (Product-C) primers, and overlapping PCR was then used to connect Product-B and Product-C with *TWI11*-FW7-Spe + *TWI11*-RV8-HA (Product-D). This overlapping PCR produced a SmaI site in the middle of the 5' flanking region. Next, Product-A and

Product-D were joined by overlapping PCR to *TWI11*-FW7-Spe and *TWI11*-RV12-Xho and inserted at the SpeI and XhoI sites of the pBlueScript SK(+) vector. Finally, the *neo3* cassette was introduced into the SmaI site. The construct was excised from the vector backbone using SpeI and XhoI and introduced into conjugating B2086 and CU428 cells by biolistic transformation. Paromomycin-resistant progeny cells were selected, and cells with the *HA-TWI11* locus in the MIC were selected by genomic PCR. Then, *HA-TWI11* loci in the MAC were removed by phenotypic assortment to obtain HA-*TWI11* heterozygous heterokaryon strains. Next, *HA-TWI11* heterozygous heterokaryon strains were mated with the "star strain" B*6 to obtain *HA-TWI11* homozygous heterokaryon (*HA-TWI11* MIC replacement) strains. The replacement of both *TWI11* loci in the MIC with the *HA-TWI11* construct was confirmed by genomic PCR with *TWI11*-MicLC-FW1 and *TWI11*-RV5-BBC.

The *TWI11* KO construct (Supplementary Figure S1I) was made by overlapping PCR. Two parts of the genomic region, including the *TWI11* gene, were amplified by PCR using *TWI11*-FW3-Spe + *TWI11*-VR5-BBC (Product-E) or *TWI11*-FW4-SCA + *TWI11*-RV4-Xho (Product-F). The *neo3* cassette was amplified by PCR using MnB-FW-BBC and MnB-RV-SCA from pNeo3. Next, Product-E, *neo3* and Product-F were joined by overlapping PCR with *TWI11*-FW3-Spe and *TWI11*-RV4-Xho. This product was introduced into conjugating B2086 and CU428 cells through biolistic transformation. Paromomycin-resistant progeny cells were selected, and cells with the *TWI11* KO locus in the MIC were chosen by genomic PCR. Then, *TWI11* KO loci in the MAC were removed by phenotypic assortment to obtain *TWI11* KO heterozygous heterokaryon strains. *TWI11* KO heterozygous heterokaryon strains were then mated with the "star strain" B*6 or

7 to obtain *TWI11* KO homozygous heterokaryon (*TWI11* MIC-KO) strains. The absence of the *TWI11* gene in the MIC was confirmed by genomic PCR with *TWI11*-MicLC-FW1 and TWI11-MicLC-RV1.

The *TWI1* MIC-KO (Mochizuki and Gorovsky, 2004) and *TWI11* MIC-KO strains were mated to obtain *TWI1/TWI11* double KO heterozygous cells. Then, *TWI1* and *TWI11* KO loci in the MAC were removed by phenotypic assortment to obtain *TWI1/TWI11* double KO heterozygous heterokaryon strains. Next, *TWI1/TWI11* double KO heterozygous heterokaryon strains were mated with the "star strain" B*6 or 7 to obtain *TWI1/TWI11* double KO homozygous heterokaryon strains (*TWI1/TWI11* double MIC-KO) strains. The absence of *TWI1* and *TWI11* genes in the MIC was confirmed through genomic PCR with *TWI1*-mic-CasMut-cFW2 and *TWI1*-mic-cRV1 or *TWI11*-MicLC-FW1 and *TWI11*-MicLC-RV1 (Supplementary Figure S1I). The DNA oligos used to make the targeting/transgenic constructs are listed in Supplementary Table 2.

To produce *MTT1*:: FLAG-HA-*TWI1* strains, which express FLAG-HA-*TWI1* from *MTT1* promoter in the MAC, FLAG-HA-*TWI1* coding sequence was amplified by PCR using the previously reported FLAG-HA-*TWI1*-WT construct (Noto et al. 2010) as template and FH-TWI1_pBMMB1_FW and FH-TWI1_pBMMB1_RV as primers. The PCR product was connected to BamHI and SpeI digested pBNMB1 (Busch et al. 2010) by Gibson Assembly kit. The resulted plasmid was the digested with XhoI and introduced into the MAC of the wild-type B2086 and CU428 strains. Phenotypic assortment was performed until the cells grew in 20-30 mg/mL of paromomycin. The strains derived from B2086 and CU428 were mated, refeeded at 3 hpm by adding SPP medium to the final concentration of

0.1x, and expression of FLAG-HA-*TWI1* was induced by adding 0.5 μg/mL CdCl2 at 5 hpm.

Antibodies

To generate the anti-Twi11p antibody, a rabbit was immunized with the fusion protein of MBP and the C-terminal half (250-798 aa) of Twi11p, expressed in *E. coli*. The rabbit polyclonal antibody for Twi1p was described previously (Aronica et al., 2008). The mouse monoclonal anti-HA antibody HA.11 clone 16B12 (Covance) is commercially available. The mouse monoclonal anti-alpha-tubulin antibody 12G10 was obtained from the Developmental Studies Hybridoma Bank at the University of Iowa.

Positions of IESs

The positions of IESs used for the analyses shown in Figure 4 in the draft MIC genome sequence are IES5: SC2.1 104,037-107,601; IES737: SC2.8 130,820-133,697; IES1147 SC2.12 1,551,243-1,553,804; IES1988: SC2.26 393,792-398,354, IES4092: SC2.80 145,860-148,725; and IES4874: SC2.110 112,688-121,910.

IESs, CDSs, TEs and Repeats distributions

The previously predicted IESs (Schoeberl et al., 2012) and manually annotated 28 IESs are listed in Supplementary Table S1. The draft MIC genome sequence (version 2) was obtained from the *Tetrahymena* Comparative Sequencing Project (Broad Institute of Harvard and MIT). The list of predicted gene CDSs (version released in October 2008 by the J. Craig Venter Institute) was obtained from the

Tetrahymena Genome Database (http://ciliate.org/index.php/home). To visualize the distributions of IESs and CDSs, their occupancies in the assembled MIC genome were calculated. Introns were also considered CDSs in this study. As representative transposable elements (TEs), two previously published *Tetrahymena* TE families, the Tlr1 and REP elements (Fillingham et al., 2004; Wuitschick et al., 2002), were used. The following sequences, obtained from GenBank, were compiled as TE sequences: AF451863; AF451864; AF451865; AF451862; AF451860.1; AF232244; F232247.1; AF232243; AF232245; AF232242.1; AF451867; AF451866; AF451869.1; AF451861.1; AF451868.1; AY371728.1; AY371729.1; AY371730.1; AY371731.2. All possible 25-nt sequences were extracted from the compiled TE sequences, and the occupancies of these 25-nt sequences in the assembled MIC genome were calculated by allowing multiple mapping to the genome. To analyze repeat frequencies, the total draft MIC genome, total Type-A IESs and total Type-B IESs were analyzed separately. See below for Type-A and Type-B IES classification. Repeats were analyzed in a manner similar to TEs, although the frequency of 25-nt sequences but not their occupancy was counted.

IES Classification

Among the annotated IESs (listed in Supplementary Table S1), 8,105 IESs longer than 300 nt and containing more than 100-nt A/C/G/T bases were used for the classification. Normalized (RPKM) and weighted (read number of each sequence was divided by the frequencies of the sequence in the draft MIC genome) numbers of small RNA reads mapping to each IES were obtained from small RNAs co-precipitated with HA-Twi11p at 10.5 hpm (=L) and oxidation-resistant

(= Twi1p-bound) small RNAs at 3 hpm (=E). The oxidation-resistant small RNAs at 3 hpm were previously described (Schoerbel et al. 2012). If L of an IES was lower than 1 RPKM, the IES was classified as a Type-C IES. If L of an IES was greater than or equal to 1 RPKM, the Late-scnRNA/Early-scnRNA index (LEI) was calculated as L/E. If E was 0, 0.01 was used for E instead. IESs with LEIs lower than 10 were defined as Type-A IESs. The remaining IESs were classified as Type-B IESs. Type-A and Type-B IESs were further divided into five subclasses: A1 (LEI ≤1); A2 (1< LEI ≤4); A3 (4<LEI ≤10); B1 (10< LEI ≤40); B2 (LEI >40).

Purification of the new MAC and DNA elimination analysis

Nuclei from exconjugants at 36 hpm were dissociated and stained with DAPI in TMSNP buffer (0.25 M sucrose, 10 mM Tris-HCl pH 7.5, 10 mM $MgCl_2$, 3 mM $CaCl_2$, 0.016% NP-40, 1 mM PMSF, 1x complete proteinase inhibitor cocktail (Roche), 0.1 μg/mL DAPI) by a dounce homogenizer with 15 strokes. The nuclear fraction was collected by centrifugation at 4,500 g at 4°C for 5 min, washed with TMSN buffer (TMSNP buffer without PMSF) 3 times, and stored at -80˚C. The nuclear fraction was resuspended with TMSN buffer containing 2 mM EDTA and 0.016 or 0.1% NP-40, and the new MACs were collected by FACSAriaIII (BD biosciences) according to size and DAPI intensity. The purity of the new Mac was assessed by immunostaining using an anti-H3K4me3 antibody (Abcam) and DAPI staining, with H3K4me3-negative nuclei counted as MICs; H3K4me3-positive, DAPI-poor small nuclei counted as new MACs; and H3K4me3-positive, DAPI-rich large nuclei counted as parental (old) MACs. Contamination with the

parental (old) MACs was rare (<1 %), and most of the contaminants in our new MAC preparations were MICs.

DNA elimination analysis with pseudo-IES

The R-IES (MIC supercontig 2.231, 124320-125409, LEI= 10.53) and its flanking sequences were amplified as two (left and right) pieces by PCR from the total genomic DNA of B2086 using the primers RfGFP_R5FW and R_Mic_clone_RV (for left) and R_Mic_clone_FW and RfGFP_R5RV (for right). These two PCR products were then connected by overlapping PCR using the RfGFP_R5FW and RfGFP_R5RV primers. To produce the "pseudo-IES," the left arm of the R-IES (20 bp of R-IES and 224 bp of the left flanking MDS) was amplified by PCR with RfGFP_R5FW and RfGFP_R5RV; the first half of the EGFP-encoding gene (Kataoka et al., 2010) was amplified with RfGFP_GFP5FW and RfGFP_GFPmRV; the second half of the EGFP-encoding gene was amplified with RfGFP_GFPmFW and RfGFP_GFPRV; and the right arm of R-IES (20 bp of R-IES and 288 bp of the right flanking MDS) was amplified by PCR with RfGFP_R5FW and RfGFP_R5RV. Then, the four PCR products were connected by overlapping PCR using the RfGFP_R5FW and RfGFP_R5RV primers. This overlapping PCR produces a NotI site in the middle of the EGFP sequence. Then, the R-IES and the pseudo-IES were digested with PspOMI and cloned into the NotI site of the extra-chromosomal (rDNA) vector pD5H8 (gift from Dr. Meng-Chao Yao, Academia Sinica, Taiwan) to produce pD5H8-R-IES and pD5H8-pseudo-IES.

coDel

M-IES (MIC supercontig 2.231: 120,715-12,1623, LEI=1.06) and its flanking sequences were amplified as two (left and right) pieces by PCR from the total genomic DNA of B2086 using the primers McoDel_5FW_PspOMI and McoDel_IESRV_Not (left) and McoDel_IESFW_Not and McoDel_3RV_PspOMI (right). Then, these two PCR products were connected by overlapping PCR using McoDel_5FW_PspOMI and McoDel_3RV_PspOMI. This overlapping PCR produced a NotI site in the middle of the IES. CL1-IES (MIC supercontig 2.218: 108,618-111,224, LEI=0.82) and CR5-IES (MIC supercontig 2.218: 40,058-46,513, LEI = 0.18) were amplified similarly using CL1coDel_5FW_PspOMI/CL1coDel_IESRV_Not/CL1coDel_IESFW_Not/CL1coDel_ 3RV_PspOMI and CR5coDel_5FW_PspOMI/CR5coDel_IESRV_Not/CR5coDel_IESFW_Not/CR5coDel_ 3RV_PspOMI, respectively. The PCR products were digested with PspOMI and cloned into the NotI site of pD5H8 to produce the pMcoDel, pCL1coDel and pCR5coDel vectors. These vectors were digested with NotI, and target sequences were inserted by Gibson Assembly (NEB). The target sequences used in this study were as follows: TTHERM_00079530 (GenBank GG662704: 1,004,795-1,005,509); TTHERM_00841280 (GenBank GG662249: 38,458-39,065); and non-coding sequence on MAC_Contig 3814 (GenBank GG662798: 1,349,433-1,350,093). These target sequences were PCR-amplified with the primer sets cDelSa1_FW/cDelSa1_RV, cDelSa18_FW/cDelSa18_RV, and cDelMC3812_FW/cDelMC3812_RV, respectively. The vectors were introduced into wild-type or *TWI11* MIC-KO cells as described in the section "DNA elimination analysis with pseudo-IES," and transformants were selected with 100 μg/mL paromomycin in 1x SPP. Total genomic DNA was extracted from

paromomycin-resistant cells using a NucleoSpin Tissue Kit (Macherey-Nagel), and DNA eliminations at the endogenous TTHERM_000795, TTHERM_00841280, and non-coding sequence on MAC_Contig 3814 loci were analyzed by PCR using TTHERM_00079530_DelCheck_FW/TTHERM_00079530_DelCheck_RV, Sa18_DelCheck_FW/Sa18_DelCheck_RV, or MC3812_DelCheck_FW/MC3812_DelCheck_RV, respectively. The primers used are listed in Table S2.

Simulation of accidental loss of Early-scnRNA expression and targeting of protein coding sequences

Among the classified Type-A, -B and -C IESs, examples longer than 500 nt and possessing more than 250 nt of A/C/G/T sequences were used as the total set of IESs (7,949 total: 4634 Type-A, 3264 Type-B, and 51 Type-C). A set of 46 (1%) or 463 (10%) non-redundant Type-A IES was randomly chosen, and all possible continuous 20-mers were extracted from the chosen Type-A IESs. Then, the extracted 20-mers were mapped to every IES. IESs containing more than 250 nt to which the 20-mers mapped in at least one of any possible continuous 500-nt segments were identified as recognized IESs (1° IESs). Then, all possible 20-mers were extracted from 1° IESs, and the same calculation was repeated to identify 2° IESs. These simulations were performed using three different sets of randomly chosen Type-A IESs for each condition (1% or 10%), and the means of the fractions of 1° and 2° IESs were calculated.

To analyze the potential targeting of the protein CDS by IES-derived scnRNAs, the predicted CDSs (see above) longer than 500 nt and possessing more than 250 nt of A/C/G/T sequences were used as the total set of CDSs

(20,491 in total). All possible continuous 20-mers were extracted from all of the predicted IESs, and the extracted 20-mers were mapped to every CDS. CDSs containing more than 250 nt to which the 20-mers were mapped in at least one of the possible continuous 500-nt segments were identified as recognized CDSs.

References for Supplemental Experimental Procedures

Aronica, L., Bednenko, J., Noto, T., DeSouza, L.V., Siu, K.W., Loidl, J., Pearlman, R.E., Gorovsky, M.A., and Mochizuki, K. (2008). Study of an RNA helicase implicates small RNA-noncoding RNA interactions in programmed DNA elimination in *Tetrahymena.* Genes & Development *22*, 2228-2241.

Fillingham, J.S., Thing, T.A., Vythilingum, N., Keuroghlian, A., Bruno, D., Golding, G.B., and Pearlman, R.E. (2004). A non-long terminal repeat retrotransposon family is restricted to the germ line micronucleus of the ciliated protozoan *Tetrahymena thermophila*. Eukaryotic Cell *3*, 157-169.

Gorovsky, M.A., Yao, M.C., Keevert, J.B., and Pleger, G.L. (1975). Isolation of micro- and macronuclei of *Tetrahymena pyriformis*. Methods in Cell Biology *9*, 311-327.

Kataoka, K., Schoeberl, U.E., and Mochizuki, K. (2010). Modules for C-terminal epitope tagging of *Tetrahymena* genes. Journal of Microbiological Methods *82*, 342-346.

Mochizuki, K., Fine, N.A., Fujisawa, T., and Gorovsky, M.A. (2002). Analysis of a *piwi*-related gene implicates small RNAs in genome rearrangement in *Tetrahymena*. Cell *110*, 689-699.

Mochizuki, K., and Gorovsky, M.A. (2004). Conjugation-specific small RNAs in *Tetrahymena* have predicted properties of scan (scn) RNAs involved in genome rearrangement. Genes & Development *18*, 2068-2073.

Mochizuki, K., and Gorovsky, M.A. (2005). A Dicer-like protein in *Tetrahymena* has distinct functions in genome rearrangement, chromosome segregation, and meiotic prophase. Genes & Development *19*, 77-89.

Noto, T., Kurth, H.M., Kataoka, K., Aronica, L., DeSouza, L.V., Siu, K.W., Pearlman, R.E., Gorovsky, M.A., and Mochizuki, K. (2010). The *Tetrahymena* argonaute-binding protein Giw1p directs a mature argonaute-siRNA complex to the nucleus. Cell *140*, 692-703.

Schoeberl, U.E., Kurth, H.M., Noto, T., and Mochizuki, K. (2012). Biased transcription and selective degradation of small RNAs shape the pattern of DNA elimination in *Tetrahymena*. Genes & Development *26*, 1729-1742.

Wuitschick, J.D., Gershan, J.A., Lochowicz, A.J., Li, S., and Karrer, K.M. (2002). A novel family of mobile genetic elements is limited to the germline genome in *Tetrahymena thermophila*. Nucleic Acids Research *30*, 2524-2537.
